# Creb5 establishes the competence for *Prg4* expression in articular cartilage

**DOI:** 10.1038/s42003-021-01857-0

**Published:** 2021-03-12

**Authors:** Cheng-Hai Zhang, Yao Gao, Unmesh Jadhav, Han-Hwa Hung, Kristina M. Holton, Alan J. Grodzinsky, Ramesh A. Shivdasani, Andrew B. Lassar

**Affiliations:** 1 Department of Biological Chemistry and Molecular Pharmacology, Blavatnik Institute at Harvard Medical School, Boston, MA USA; 2 Department of Medical Oncology and Center for Functional Cancer Epigenetics, Dana-Farber Cancer Institute, Boston, MA USA; 3 Departments of Medicine, Brigham & Women’s Hospital and Harvard Medical School, Boston, MA USA; 4 Department of Biological Engineering, Massachusetts Institute of Technology, Cambridge, MA USA; 5 Research Computing, Harvard Medical School, Boston, MA USA; 6 Harvard Stem Cell Institute, Cambridge, MA USA

**Keywords:** Molecular biology, Stem cells, Developmental biology

## Abstract

A hallmark of cells comprising the superficial zone of articular cartilage is their expression of lubricin, encoded by the *Prg4* gene, that lubricates the joint and protects against the development of arthritis. Here, we identify Creb5 as a transcription factor that is specifically expressed in superficial zone articular chondrocytes and is required for TGF-β and EGFR signaling to induce *Prg4* expression. Notably, forced expression of Creb5 in chondrocytes derived from the deep zone of the articular cartilage confers the competence for TGF-β and EGFR signals to induce *Prg4* expression. Chromatin-IP and ATAC-Seq analyses have revealed that Creb5 directly binds to two *Prg4* promoter-proximal regulatory elements, that display an open chromatin conformation specifically in superficial zone articular chondrocytes; and which work in combination with a more distal regulatory element to drive induction of *Prg4* by TGF-β. Our results indicate that Creb5 is a critical regulator of *Prg4*/lubricin expression in the articular cartilage.

## Abstract (web summary)

Zhang et al. identify Creb5 as a transcription factor that is required for induction of *Prg4* expression by TGFβ and EGFR ligands. They show that Creb5 directly binds to two *Prg4* promoter-proximal regulatory elements, working together with a more distal regulatory element to drive induction of *Prg4* by TGFβ. These findings provide new insight into the molecular regulation of *Prg4* expression in superficial zone articular chondrocytes.

## Introduction

Osteoarthritis (OA) affects over 30 million US adults (CDC statistics). Interventions that block the progression of OA are currently unknown. With an eye toward this goal, we have sought to develop a comprehensive understanding of the regulatory network that regulates the differentiation and maintenance of articular cartilage, which plays a central role in maintaining the low-friction environment of the joint space. A hallmark of cells comprising the articular cartilage is their expression of proteoglycans, such as the protein lubricin, encoded by the *Prg4* gene, that lubricates the joint and protects against the development of OA^1,2^. *Prg4* is specifically expressed in the superficial-most layer of the articular cartilage, but not by deeper layers of this tissue^2–7^. Fate mapping studies have established that *Prg4*-expressing cells in embryonic and early postnatal joints constitute a progenitor pool for all regions of the articular cartilage in the adult^8–10^. These findings are consistent with prior studies indicating that both superficial and deep zones of the articular cartilage (plus other synovial joint tissues) specifically arise from *Gdf5*-expressing cells in the embryo^11–13^. In both humans and mice lacking *Prg4*, the surface of the articular cartilage becomes damaged and precocious joint failure occurs^1,2^. Notably, decreased levels of lubricin have been observed in the synovial fluid following either surgically induced OA in sheep^14^, in human synovial fluid samples from patients with either OA or rheumatoid arthritis^15^, and in the menisci from OA patients^16^. Furthermore, a decrease in *Prg4*/lubricin expression during aging^17^ correlates with increasing sensitivity of aged knees to cartilage degradation following knee joint destabilization^18^. Most notably, loss of lubricin (in *Prg4*^*−/−*^ mice) has been noted to result in significantly higher levels of peroxynitrite, superoxide, and cleaved caspase 3^19^, which correlates with increased levels of both whole-joint friction and cellular apoptosis in *Prg4*^*−/−*^ mice compared with either wild-type or *Prg4*^*+/−*^ mice^20^. Indeed, a hallmark of both aging^21,22^ and OA^23^ is a loss of cells in the superficial zone of the articular cartilage. Conversely, if lubricin protein is either injected directly into the synovial fluid^24–28^ or overexpressed in the knee joint (via transgene or AAV;^29,30^) the articular cartilage tissue is protected from degradation following surgically induced joint destabilization. Taken together, these findings indicate that *Prg4*/lubricin counters the signaling pathways that lead to cartilage destruction and suggest that identifying a means to induce the sustained expression of *Prg4* in the articular cartilage may attenuate the degradation of articular cartilage observed during either aging or OA.

While Foxo^31^, Nfat^32^, and Creb1^33^ transcription factors (TFs) have been found to modulate *Prg4* expression in articular chondrocytes, the TFs that drive either tissue-specific expression of *Prg4* or region-specific expression of this gene in the superficial zone of articular cartilage have not yet been elucidated. In addition, as *Prg4* is expressed in articular chondrocytes but not in growth plate chondrocytes, we speculated that identification of the TFs that control expression of this gene in the superficial zone of articular cartilage may elucidate how these distinct chondrocyte cell fates are regulated. Several signaling pathways, including Wnt^11,34,35^, TGF-β ^36,37^, and EGFR^38^, have all been found necessary to maintain the expression of *Prg4* in the superficial zone of articular cartilage. Interestingly, however, injurious mechanical compression^39^, TGF-β1 exposure^36^, and shear stress from fluid flow^33^ can induce *Prg4* expression exclusively in explants taken from the superficial zone of bovine articular cartilage, and not in explants from middle or deep zones. This highly restricted induction implies that superficial zone cells either secrete additional necessary signals or uniquely express a TF(s) that responds to the inductive signals. Here, we identify Creb5 as a TF that is specifically expressed in both bovine and human superficial zone articular cartilage and is critically required to activate *Prg4* expression, in response to TGF-β and EGFR signaling.

## Results

### Creb5, a TF expressed selectively in *Prg4*^*+*^ chondrocytes

**Fig. 1:**
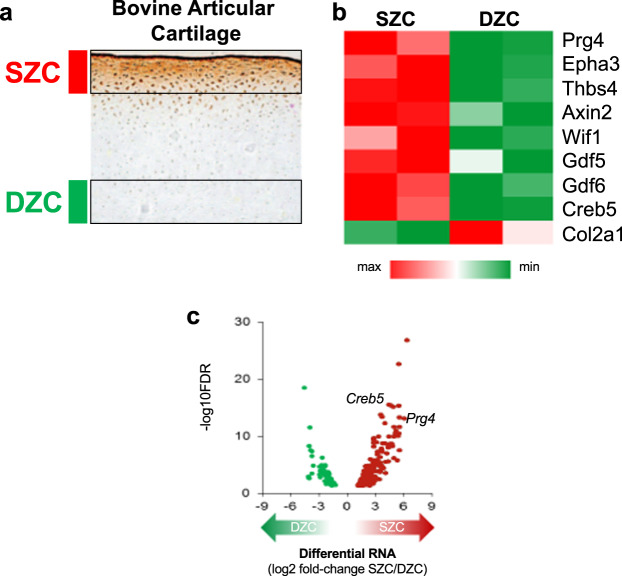
Identification of genes that are differentially expressed in superficial versus deep zone articular chondrocytes. **a** Lubricin (brown) is expressed in superficial zone chondrocytes (SZCs; adjacent to red bar) and not in deep zone chondrocytes (DZCs; adjacent to green bar) in bovine articular cartilage. Image of lubricin immunostaining taken from^44^. **b** Relative expression of genes that are differentially expressed in superficial zone bovine articular chondrocytes (SZC) versus deep zone bovine articular chondrocytes (DZC). Maximal and minimal expression levels in SZCs and DZCs are indicated by the intensity of the red and green hues, respectively. **c** Volcano plot of differentially expressed genes (DEGs) in SZCs and DZCs. Each dot represents one gene. The red dots represent SZC-specific DEGs, the green dots represent DZC-specific DEGs. Note that the TF gene *Creb5* was as differentially expressed as *Prg4*, with ~25-fold higher levels in SZCs than in DZCs.

**Fig. 2:**
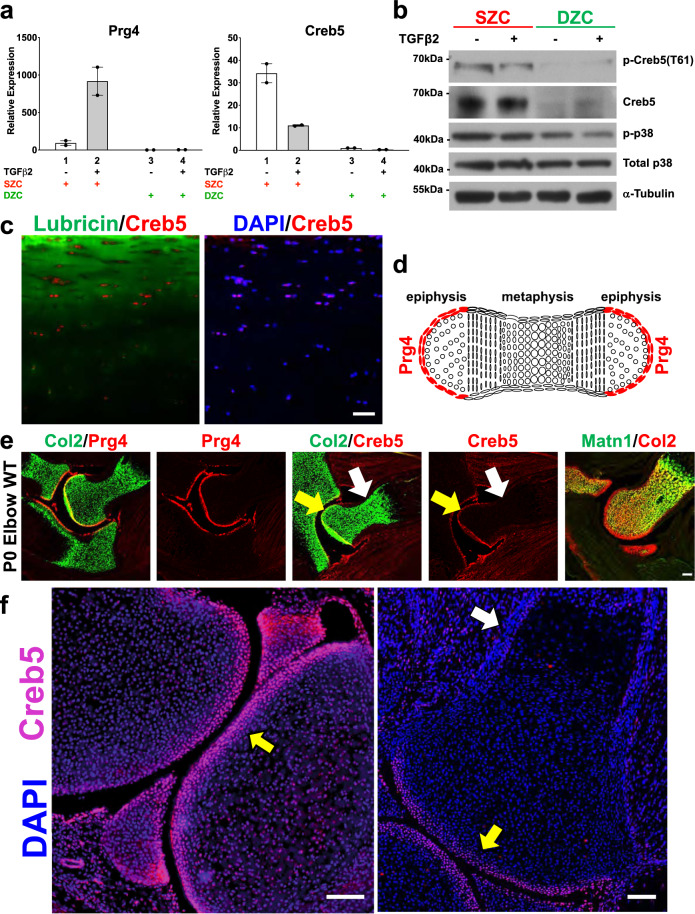
The transcription factor Creb5 is differentially expressed in superficial versus deep zone articular chondrocytes and is specifically expressed in *Prg4*^*+*^ cells in synovial joints. **a** RT-qPCR analysis of *Prg4* and *Creb5* expression in either SZCs or DZCs cultured in either the absence (white) or presence (gray) of TGF-β2 (20 ng/ml) for 3 days. Gene expression was assayed by RT-qPCR and normalized to *Gapdh*. Error bar indicates standard error of the mean (*n* = 2 technical repeats). Similar results have been obtained in three independent biological repeats. **b** Western analysis of proteins in SZCs and DZCs. Similar results have been obtained in two independent biological repeats. **c** Immunofluorescent staining for CREB5, lubricin, and DAPI (to visualize nuclei) in adult human femoral head articular cartilage. Scale bar equals 50 microns. **d** Schematic of *Prg4* expression in a developing long bone cartilage element. **e** Expression of *Collagen 2a1 (Col2), Prg4, Creb5*, and *Matrilin1 (Matn1)* in the elbows of P0 mice, as detected by fluorescent in situ hybridization. **f** Immunofluorescent staining for Creb5 protein and DAPI (to visualize nuclei) in the knee joint of a P0 mouse. In (**e**, **f**), the Creb5-expressing articular perichondrium is designated by a yellow arrow, the metaphyseal perichondrium is designated by a white arrow; and the scale bars equal 100 microns.

In newborn bovine knee joint cartilage, superficial zone chondrocytes (SZCs) expressing *Prg4/*lubricin are readily distinguished from *Prg4/*lubricin*-*negative deep zone chondrocytes (DZCs, Fig. 1a). As the profound differences between these two tissues likely have a transcriptional basis, we employed RNA-Seq to identify SZC-specific TF genes. Reflecting their common developmental origin^8–10^, SZCs and DZCs differed by only 320 genes (Supplementary Data 1; false discovery rate, FDR < 0.05), including ~67-fold higher *Prg4* mRNA levels in SZCs, as expected, and >15-fold higher levels of other SZC marker genes such as *Vitrin*, *Epha3*, *Wif1*, and *Thbs4* (Supplementary Data 1; Fig. 1b). Notably, we found that 29 transcriptional regulators were more highly expressed (at least twofold) in SZCs than in DCZs; and only one TF (Sall1) was more highly expressed in DZCs (Table 1). The TF gene *Creb5* was the most differentially expressed transcriptional regulator, with ~25-fold higher levels in SZCs than in DZCs (as determined by RNA-Seq; Fig. 1c), approximately equal to the differential expression of *Prg4* in these tissues. Because TGF-β induces *Prg4* specifically in SZCs in vitro^36^ and maintains *Prg4* expression in articular cartilage in vivo^37^, we assessed mRNA levels in SZCs and DZCs cultured with or without TGF-β2. RT-qPCR revealed approximately tenfold induction of *Prg4* by TGF-β2 only in SZCs, as others have reported^36^; and a selective 250-fold (*Prg4*) and 40-fold (*Creb5*) greater expression of these transcripts, in response to TGF-β2, in SZCs versus DZCs (Fig. 2a, compare lanes 2 and 4).

**Table 1:** Differentially expressed regulators of gene expression.

GeneSymbol	Ensembl	logFC	FDR
Superficial zone enriched regulators of gene expression			
CREB5	ENSBTAG00000018909	4.626876	4.39E-16
NRIP3	ENSBTAG00000013366	3.322989	6.63E-08
ZFPM2	ENSBTAG00000001649	3.13596	1.33E-09
HOPX	ENSBTAG00000002333	2.985838	1.94E-06
TSHZ2	ENSBTAG00000007917	2.838663	4.46E-06
HOXD3	ENSBTAG00000004835	2.64649	0.001064
DLX3	ENSBTAG00000017409	2.618468	0.000391
NR4A3	ENSBTAG00000001864	2.586009	7.92E-05
HOXD4	ENSBTAG00000039581	2.429548	6.68E-05
TBX5	ENSBTAG00000011384	2.303632	0.000289
PEG3	ENSBTAG00000023338	2.278346	0.000484
TBX18	ENSBTAG00000018161	2.129558	0.041923
ETV5	ENSBTAG00000014915	2.121456	0.004447
TBX15	ENSBTAG00000007767	2.035948	8.00E-05
OSR2	ENSBTAG00000013213	2.016969	0.000634
NR4A2	ENSBTAG00000003650	2.013499	0.001064
HDAC9	ENSBTAG00000003808	1.885373	0.001849
GLI3	ENSBTAG00000010671	1.766052	0.004555
ERG	ENSBTAG00000011001	1.763597	0.000435
NFATC2	ENSBTAG00000018270	1.759048	0.0142
NFKBIZ	ENSBTAG00000010987	1.674727	0.044988
NFIB	ENSBTAG00000027442	1.562924	0.003388
NR4A1	ENSBTAG00000000507	1.53171	0.00366
HR	ENSBTAG00000012036	1.459785	0.013929
DACT1	ENSBTAG00000019421	1.421286	0.02876
ZFP36L1	ENSBTAG00000025434	1.327187	0.031884
TBX4	ENSBTAG00000009968	1.326603	0.023106
ZNF609	ENSBTAG00000015808	1.265181	0.039834
ZNF385C	ENSBTAG00000046162	1.257806	0.038465
Deep zone enriched regulators of gene expression			
SALL1	ENSBTAG00000008544	−2.62385	0.000294

Transcriptional regulators are listed whose expression differed (by indicated LogFC) between superficial zone articular chondrocytes and deep zone articular chondrocytes (false discovery rate, FDR < 0.05).

**Fig. 3:**
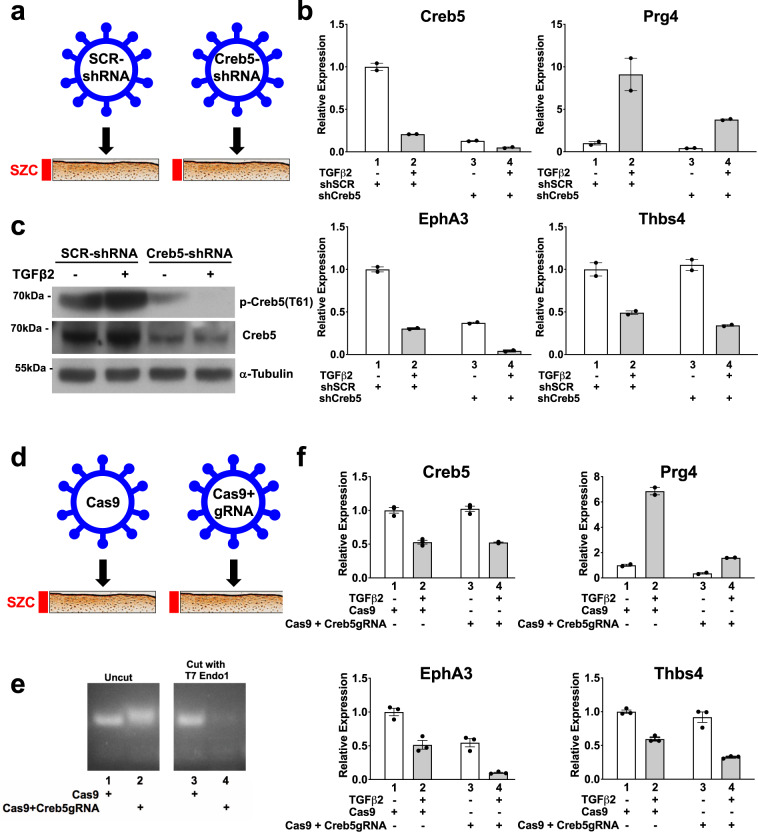
Creb5 function is necessary for TGF-β-dependent induction of *Prg4* in superficial zone articular chondrocytes. **a**–**c** shRNA-mediated knock-down of Creb5 in superficial zone articular chondrocytes. SZCs were infected with a lentivirus encoding either control scrambled shRNA (shSCR, lanes 1 and 2) or shCreb5 (lanes 3 and 4), after selection in puromycin, the cells were cultured in either the absence (white) or presence (gray) of TGF-β2 (20 ng/ml) for 3 days. **b** Gene expression was assayed by RT-qPCR and normalized to *Gapdh*. Error bar indicates standard error of the mean (*n* = 2 technical repeats). Similar results have been obtained in three independent biological repeats. **c** Western analysis of proteins in SZCs recognized by antibodies directed against either phospho-Creb5 (T61), total Creb5, or α-tubulin. Similar results have been obtained in two independent biological repeats. **d**–**f** CRISPR-Cas9 mediated mutation of the DNA-binding domain of *Creb5* in SZCs. SZCs were infected with a lentivirus encoding Cas9 alone or Cas9 plus a *Creb5* guide RNA (targeting the DNA-binding domain), as indicated. After selection in puromycin, the cells were cultured in either the absence (white) or presence (gray) of TGF-β2 (20 ng/ml) for 3 days. **e** T7 Endonuclease 1 assay (which cleaves at mismatches) is displayed for the RT-PCR amplicon encoding the bZIP domain of *Creb5*. **f** Gene expression was assayed by RT-qPCR and normalized to *Gapdh*. Error bar indicates standard error of the mean (*n* = 3 technical repeats). Similar results have been obtained in two independent biological repeats.

The basic-leucine zipper (bZIP) DNA-binding domain of Creb5 shares high sequence homology with those of Atf2^40^ and Atf7^41^. In addition, all three TFs carry two conserved N-terminal Threonine/Proline residues (T59 and T61 in Creb5) that are substrates for the P38, Jun N-terminal (JNK), and extracellular signal-regulated (ERK) kinases (reviewed in^42,43^). Consistent with selective *Creb5* mRNA expression in SZCs, a ~65-kDa protein recognized by both Creb5 and phospho-specific Creb5 (T61) antibodies is enriched specifically in SZCs (Fig. 2b). Infection of SZCs with lentivirus encoding an shRNA directed against the 3′UTR of *Creb5* substantially diminished these protein levels (Fig. 3c). Of note, phospho-P38 kinase, the active form that can phosphorylate Creb5 on T61, is also more abundant in SZCs than in DZCs (Fig. 2b). Although TGF-β2 diminished the level of *Creb5* mRNA to ~30% of that in untreated SZCs (Fig. 2a, lanes 1 and 2), this decrease had no appreciable effect on either total or phospho-Creb5 protein levels (Figs. 2b and 3c). Consistent with the restricted expression of *Creb5* in the superficial zone of bovine articular cartilage, we similarly detected nuclear-localized Creb5 specifically in the lubricin-expressing superficial zone of articular cartilage in adult human femoral head tissue (Fig. 2c).

*Prg4* is initially expressed in the articular perichondrium, which encases the epiphyses of the developing long bones (depicted schematically in Fig. 2d); and is subsequently expressed in the superficial-most layer of mature articular cartilage, but not by deeper layers of this tissue^2–7^. We used RNA in situ hybridization to localize *Creb5* transcripts in relation to *Prg4* and *Collagen 2a1 (Col2a1)* in the elbow joints of newborn mice. Consistent with our findings in bovine articular chondrocytes, we detected *Creb5* transcripts in *Prg4*-expressing cells (Fig. 2e). More specifically, *Creb5* expression was restricted to the articular perichondrium (Fig. 2e, yellow arrow), where Prg4^+^ precursor cells are known to generate articular cartilage^8–10^, and was absent from perichondrial cells adjacent to the nascent metaphyseal growth plates of developing long bones (Fig. 2e, white arrow). While articular chondrocytes express *Col2a1* but not *Matrilin1 (Matn1)*, epiphyseal chondrocytes (which will later undergo endochondral ossification) express both *Matn1* and *Col2a1*^10^. Notably, expression of *Creb5* (and *Prg4*) is restricted to the articular (Col2a1^+^/Matn1^−^) chondrocytes (Fig. 2e). In developing mouse knees, *Prg4* is expressed in the articular perichondrium, in superficial cells of the prospective meniscus, and in synovial fibroblasts that line the joint cavity^2,4,44^. Creb5 immunocytochemistry on newborn mouse knees indicated that Creb5 protein was specifically expressed and nuclear localized in these very regions (Fig. 2f, yellow arrow designates the articular perichondrium) and was absent from the metaphyseal perichondrium adjacent to the growth plate (Fig. 2f, white arrow). Thus, Creb5 co-localizes precisely with cells that express *Prg4* in newborn bovine and murine joints, and in adult human articular cartilage.

### Creb5 is necessary for induction of *Prg4* by TGF-β

To examine the role of Creb5 in regulating SZC-specific genes in articular chondrocytes, we infected primary bovine SZCs with a control lentivirus (encoding puromycin resistance and carrying a shSCR) or one engineered to express an shRNA directed against the *Creb5* 3′UTR (Fig. 3a–c). After selection in puromycin, we cultured the cells for 3 additional days with or without TGF-β2, in ultra-low attachment dishes to induce a round cell shape, which favors chondrogenic differentiation^45^. The shRNA against the *Creb5* 3′UTR attenuated *Creb5* transcripts (Fig. 3b) and protein (Fig. 3c), and reduced TGF-β2 induction of *Prg4* by 58% (Fig. 3b; compare lanes 2 and 4). Because shRNAs can have off-target effects^46^, we also infected primary SZCs with lentivirus encoding puromycin resistance and Cas9 either without (control) or with a guide RNA targeting cleavage within the *Creb5* DNA-binding domain (Fig. 3d–f). After selection in puromycin, the cells were cultured for an additional 14 days, to increase mutagenesis of the *Creb5* gene. A T7 Endonuclease 1 assay^47^ confirmed efficient introduction of indels in the targeted bZIP domain of *Creb5* (Fig. 3e). While 33% of indels (insertion/deletions) induced by CRISPR/Cas9 mutagenesis are predicted to generate mutations within the bZIP domain that still maintain the reading frame for Creb5; the remaining 67% will produce out of frame proteins. Indeed, infection of SZCs with lentivirus encoding Cas9 plus the *Creb5* gRNA resulted in decreased levels of Creb5 protein (Supplementary Fig. 1). Consistent with the notion that Creb5 DNA-binding activity is necessary for TGF-β dependent expression of *Prg4*, TGF-β2 induction of *Prg4* was reduced by 77% in SZCs containing indels in the *Creb5* bZIP domain (Fig. 3f, compare lanes 2 and 4). Notably, the level of *Prg4* expression induced in SZCs by TGF-β2 was proportional to the residual level of Creb5 protein expressed in these cells, following mutation of the Creb5 DNA-binding domain (Supplementary Fig. 1). Among the handful of other SZC-specific genes whose expression we assayed by RT-qPCR, either shRNA-mediated knock-down of *Creb5* or CRISPR/Cas9-generated indels in the bZIP domain of *Creb5* decreased baseline expression of *Epha3*, but not that of *Thbs4* (Fig. 3b, f; compare lanes 1 and 3). Interestingly, however, in the presence of TGF-β2, either shRNA-mediated knock-down of *Creb5* or loss of Creb5 DNA interaction decreased the levels of both *Epha3* and *Thbs4* (Fig. 3b, f; compare lanes 2 and 4). As TGF-β2 administration reduced *Creb5* transcript levels but not Creb5 protein levels, it is possible that this treatment both destabilizes pre-existing RNAs in SZCs (such as *Creb5, Epha3*, and *Thbs4*) while inducing the expression of *Prg4*, which requires this signal for its expression^36,37^. Taken together, these findings suggest that Creb5 is necessary to both maintain expression of some SZC-specific genes (i.e., *Epha3* and *Thb4*) in the presence TGF-β signaling; and is essential for TGF-β signals to induce maximal *Prg4* expression.

### Creb5 confers competence for *Prg4* expression in DZCs

**Fig. 4:**
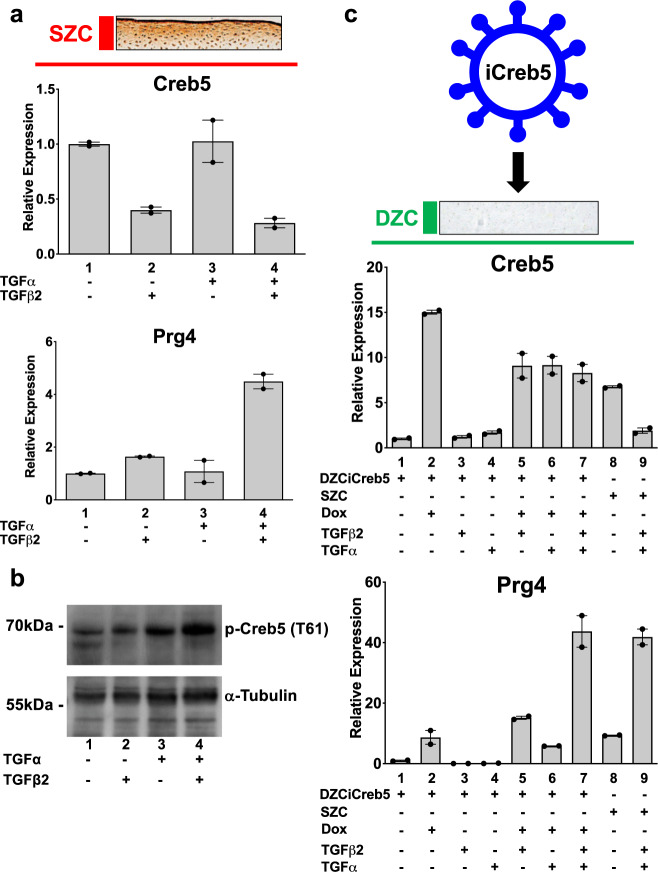
Forced expression of Creb5 in deep zone articular chondrocytes confers competence for EGFR and TGF-β signals to induce expression of *Prg4*. **a**, **b** EGFR and TGF-β signals synergistically induce expression of *Prg4* in SZCs. SZCs were treated for 2 days with TGF-β2 (20 ng/ml) and TGF-α (100 ng/ml), as indicated. **a** Gene expression was assayed by RT-qPCR and normalized to *Gapdh*. Error bar indicates standard error of the mean (*n* = 2 technical repeats). Similar results have been obtained in three independent biological repeats. **b** Western analysis of proteins in SZCs cultured in either the absence or presence of TGF-β2 (20 ng/ml) and TGF-α (100 ng/ml). Similar results have been obtained in two independent biological repeats. **c** Forced expression of Creb5 in DZCs promotes synergistic induction of *Prg4* by TGF-α and TGF-β2. DZCs were infected with a lentivirus encoding doxycycline-inducible Creb5. After selection in G418, the cells were cultured in either the absence or presence of doxycycline (1 μg/ml), TGF-β2 (20 ng/ml) and TGF-α (100 ng/ml), as indicated for 3 days. Gene expression was assayed in both the iCreb5-DZCs (lanes 1–7) and in control SZCs (lanes 8 and 9) by RT-qPCR and normalized to *Gapdh*. Error bar indicates standard error of the mean (*n* = 2 technical repeats). Similar results have been obtained in three independent biological repeats.

**Fig. 5:**
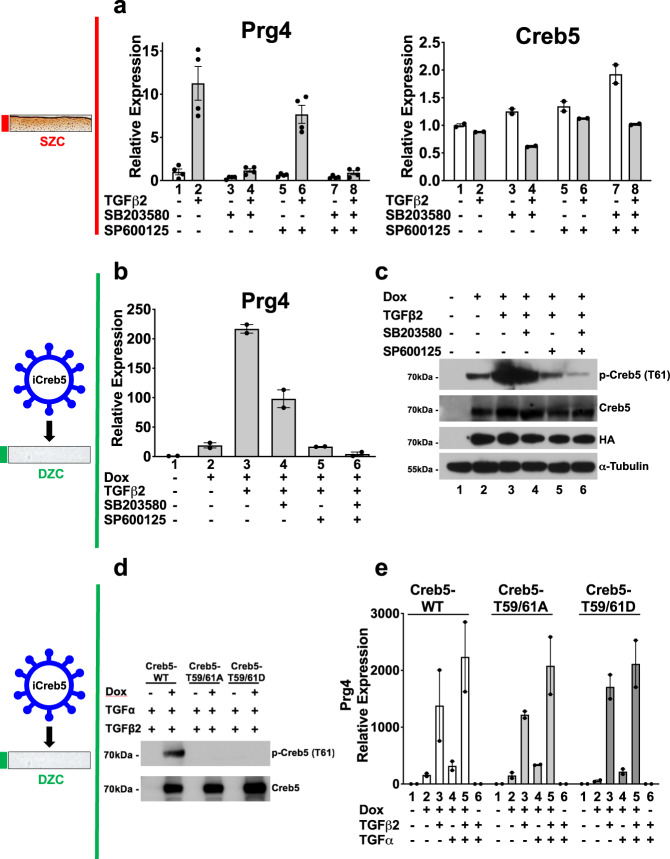
Creb5-dependent induction of Prg4 requires SAPK activity, but neither phosphorylation of T59 nor T61 in Creb5. **a** TGF-β2 induced expression of *Prg4* in superficial zone articular chondrocytes is blocked by inhibition of p38 activity. SZCs were cultured in either the absence (white) or presence (gray) of TGF-β2 (20 ng/ml), plus a p38 inhibitor (SB203580; 10 μM), or a JNK inhibitor (SP600125; 10 μM), as indicated for 2 days. Gene expression was assayed by RT-qPCR and normalized to *Gapdh*. Error bar indicates standard error of the mean (*n* = 4 technical repeats for *Prg4* expression, and *n* = 2 technical repeats for *Creb5* expression). Similar results have been obtained in three independent biological repeats. **b**, **c** DZCs were infected with a lentivirus encoding doxycycline-inducible Creb5-HA. After selection in G418, the cells (iCreb5-DZCs) were cultured in either the absence or presence of doxycycline (1 μg/ml), TGF-β2 (20 ng/ml), a p38 inhibitor (SB203580; 10 μM), or a JNK inhibitor (SP600125; 10 μM), as indicated for 2 days. **b** Gene expression was assayed by RT-qPCR and normalized to *Gapdh*. Error bar indicates standard error of the mean (*n* = 2 technical repeats). Similar results have been obtained in three independent biological repeats. **c** Western analysis of exogenous Creb5-HA expression and phosphorylation in iCreb5-DZCs with antibodies directed against either phospho-Creb5 (T61), total Creb5, HA, or α-tubulin. Similar results have been obtained in two independent biological repeats. **d**, **e** DZCs were infected with a lentivirus encoding either iCreb5-WT-HA; iCreb5-T59,61A-HA; or iCreb5-T59,61D-HA. After selection in G418, the cells were cultured in either the absence or presence of doxycycline (1  μg/ml), TGF-β2 (20 ng/ml), and TGF-α (100 ng/ml) as indicated for 3 days. **d** Western analysis of iCreb5-WT/mutant expression in iCreb5-DZCs with antibodies directed against either phospho-Creb5 (T61) or total Creb5. Similar results have been obtained in two independent biological repeats. **e** Gene expression was assayed by RT-qPCR and normalized to *Gapdh*. Error bar indicates standard error of the mean (*n* = 2 technical repeats). Similar results have been obtained in three independent biological repeats.

As both TGF-β^36,37^ and EGFR^38^ signaling promote *Prg4* expression in articular cartilage, we asked if these pathways might regulate *Creb5* expression or phosphorylation. Treatment of primary bovine SZCs with TGF-β2, but not the EGFR agonist TGF-α, reduced *Creb5* mRNA (Fig. 4a, lanes 2 and 3). However, in SZCs, the combination of TGF-β2 and TGF-α synergistically boosted both *Prg4* expression (Fig. 4a, lane 4) and phospho-Creb5 (T61) levels (Fig. 4b). Because the highly biased expression of Creb5 in SZCs versus DZCs correlates with the restricted ability of TGF-β to induce *Prg4* expression in superficial zone cells (Fig. 2a), we next asked whether Creb5 is the key factor distinguishing the competence for *Prg4* induction in SZCs versus DZCs. To this end, we infected DZCs with a pInducer20 lentivirus^48^ engineered to express a doxycycline-inducible *Creb5* cDNA appended with three carboxy-terminal hemagglutinin epitope tags (iCreb5-HA). Indeed, treatment of iCreb5-HA infected DZCs (iCreb5-DZCs) with doxycycline, TGF-β2 and TGF-α boosted *Prg4* expression ~44-fold (Fig. 4c, compare lanes 1 and 7), equal to *Prg4* levels in SZCs treated with the same ligands (Fig. 4c, lane 9). In contrast, iCreb5-DZCs treated with TGF-β2 and TGF-α failed to activate *Prg4* in the absence of doxycycline (Fig. 5e, lane 6). Thus, forced expression of Creb5 in DZCs is sufficient to promote the competence for *Prg4* induction by EGFR and TGF-β signals.

In the limbs of newborn mice, expression of both *Creb5* and *Prg4* is robust in articular chondrocytes and is absent from growth plate chondrocytes (Fig. 2f). To begin to determine whether *Creb5* can confer competence for *Prg4* expression in growth plate-like chondrocytes, we infected either a human chondrosarcoma cell line (SW1353) or an immortalized human costal chondrocyte cell line (C-28/I2;^49^) with lentivirus encoding either EGFP or Creb5. Notably, forced expression of Creb5 conferred competence for TGF-β signaling to induce *PRG4* expression in both cell lines (Supplementary Fig. 2). However, in contrast to deep zone bovine articular chondrocytes, in which the combination of *Creb5* and TGF-β signaling promotes relatively high-level expression of *Prg4* (approximately equal to 75% of *Gapdh* transcript levels), in the human chondrogenic cell lines, the combination of *Creb5* and TGF-β signaling only induced *PRG4* expression to ~0.2% of *GAPDH* transcript levels. Taken together, these findings indicate that *Creb5* can confer competence for *Prg4* induction; and suggest that this TF may work together with other factors in articular chondrocytes to promote high-level expression of *Prg4* in response to TGF-β signals.

### Creb5-dependent induction of *Prg4* requires SAPK activity

Because TGF-β2 and TGF-α synergistically boosted both *Prg4* expression and Creb5 (T61) phosphorylation, we asked whether stress-activated protein kinases (SAPKs) are necessary to induce *Prg4* expression. In SZCs, TGF-β2 induction of *Prg4* was specifically blocked by SB203580, an inhibitor of p38 kinase, but not by SP600125, a JNK antagonist (Fig. 5a); p38 inhibition altered *Creb5* transcript levels only slightly (Fig. 5a). In iCreb5-DZCs, induction of *Prg4* by TGF-β2 was partially blocked by both inhibitors, and completely blocked by the combination (Fig. 5b). Thus, SAPKs are necessary to promote *Prg4* expression by either endogenous or exogenous Creb5 in chondrocytes. In addition, the combination of SAPK inhibitors decreased iCreb5 phosphorylation on T61 (Fig. 5c). To clarify whether SAPK phosphorylation of T59 and T61 on Creb5 is necessary for *Prg4* induction, we mutated both these residues to alanine to block phosphorylation, or to aspartic acid to mimic constitutive phosphorylation. The transcriptional activity of a chimeric protein containing the GAL4 DNA-binding domain fused to the N-terminus of Creb5 (GAL4-Creb5 (1-128)) was attenuated by simultaneous T59A/T61A mutations, suggesting that phosphorylation of these residues may indeed promote Creb5 transcriptional activity (Supplementary Fig. 3). Interestingly, however, a chimeric protein containing full length Creb5 (GAL4-Creb5 (1-508)) drove greater target gene expression than a chimeric protein containing only the N-terminus of Creb5 (GAL4-Creb5 (1-128)), suggesting that regions outside the N-terminus of this protein can also drive transcriptional activation (Supplementary Fig. 3). As anticipated, phospho-Creb5 (T61) antibody failed to recognize both mutant forms of Creb5 (Fig. 5d). Surprisingly, however, in response to TGF-β2 and/or TGF-α, both mutant iCreb5 forms induced *Prg4* expression to the same level as did wild-type iCreb5 (Fig. 5e). Thus, although SAPKs promote Creb5 T61 phosphorylation, their requirement for *Prg4* induction must depend on phosphorylation either of other proteins, or of Creb5 sites other than T59 and T61.

### Identification of *Prg4* regulatory elements

**Fig. 6:**
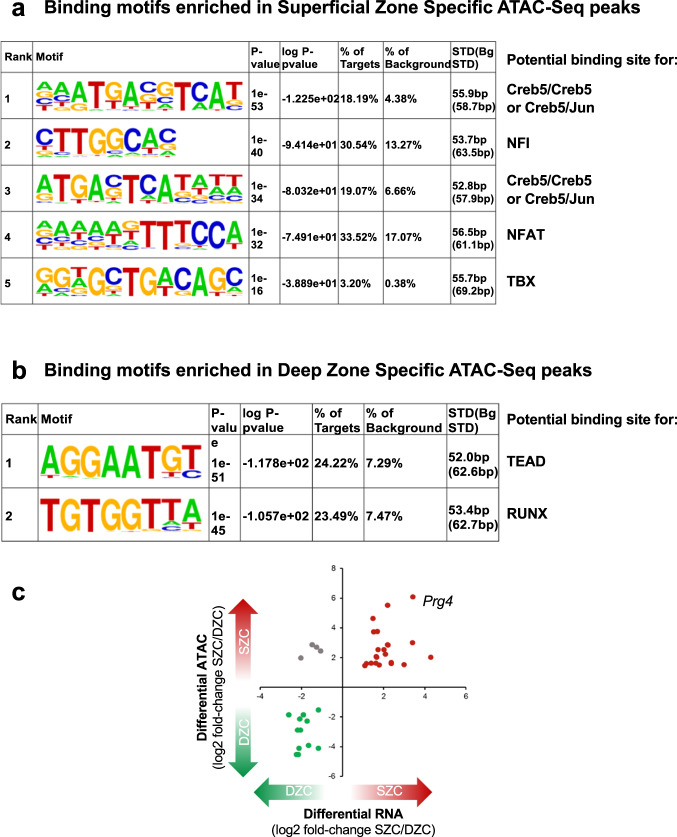
Transcription factor binding sites that are enriched in ATAC-Seq peaks which display differential accessibility in superficial versus deep zone chondrocytes. **a** Homer motif analysis of ATAC-Seq peaks that are enriched in SZCs versus DZCs indicated that binding sites for Creb5, Nfi, Nfat, and Tbx TFs are enriched in superficial zone-specific ATAC-Seq peaks. **b** Homer motif analysis of ATAC-Seq peaks that are enriched in DZCs versus SZCs indicated that the binding sites for Tead and Runx TFs are enriched in deep zone-specific ATAC-Seq peaks. In both (**a**, **b**), the frequency of these sequence motifs in zone-specific ATAC-Seq peaks (% of targets) versus their frequency in the genome (% background) is displayed. **c** Correlation between ATAC-Seq sites and zone-specific gene expression. A high correlation was observed between zone-specific ATAC-Seq sites (located ≤ 25 kb from the transcription start site) and zone-specific gene expression in both SZCs and DZCs. This correlation was highest at the *Prg4* locus, where four distinct regions (E1–E4) were selectively accessible in SZCs.

**Fig. 7:**
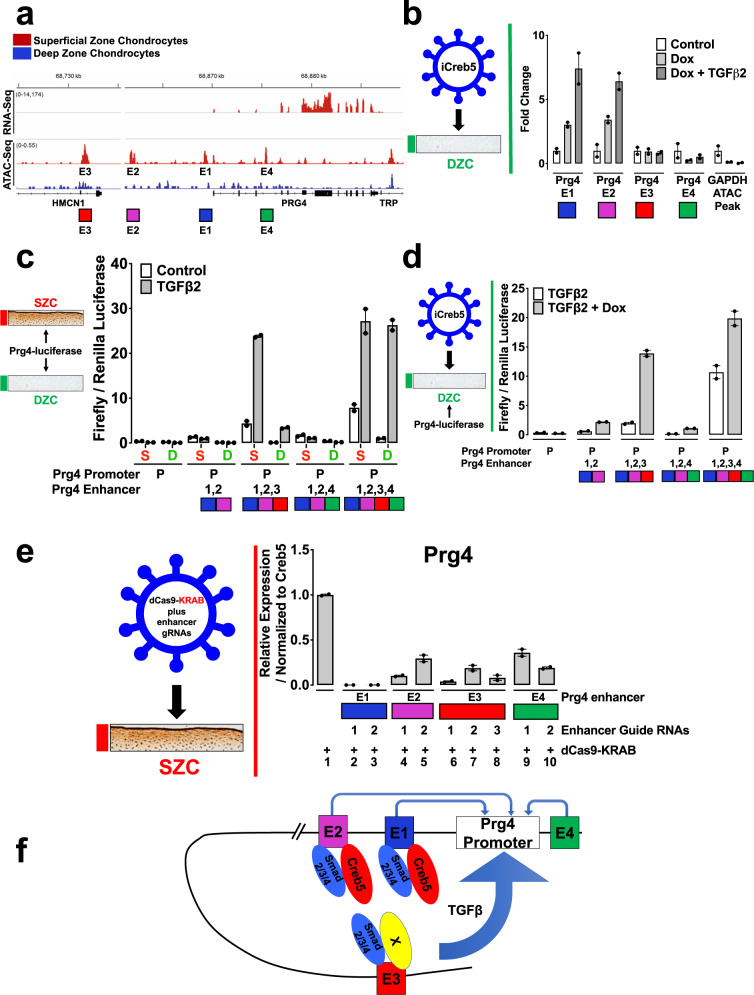
*Prg4* expression is regulated by the combinatorial activity of several regulatory elements. **a** Comparison of RNA-Seq (top tracks) and ATAC-Seq (bottom tracks) surrounding the *Prg4* locus. Signals for either SZCs (red) or DZCs (blue) are displayed. Putative *Prg4* regulatory elements (E1–E4) are indicated. **b** Bovine articular chondrocytes were infected with lentivirus encoding doxycycline-inducible HA-tagged Creb5 (iCreb5-HA). The cells were cultured in either the absence or presence of doxycycline and TGF-β2, as indicated. ChIP-qPCR was performed with anti-HA. Creb5-HA occupancy on ATAC-Seq peaks E1–E4 in the *Prg4* locus or in the *Gapdh* locus are displayed. Error bar indicates standard error of the mean (*n* = 2 technical repeats). Similar results have been obtained in two independent biological repeats. **c** SZC-enriched ATAC-Seq peaks surrounding the *Prg4* locus work in combination. Either superficial zone (S) or deep zone (D) bovine articular chondrocytes were co-transfected with a firefly luciferase reporter driven by both the *Prg4* promoter plus a combination of enhancers (E1–E4) surrounding this gene and a CMV-renilla luciferase construct. The cells were cultured in either the absence or presence of TGF-β2, as indicated. Relative expression of firefly/renilla luciferase is displayed. Error bar indicates standard error of the mean (*n* = 2 technical repeats). Similar results have been obtained in three independent biological repeats. **d** DZCs were infected with a lentivirus encoding doxycycline-inducible Creb5-HA. After selection in G418, the cells (iCreb5-DZCs) were transfected with Prg4-firefly luciferase expression constructs as described above and cultured with TGF-β2 (20 ng/ml) in either the absence or presence of doxycycline (1 μg/ml), as indicated for 2 days. Relative expression of firefly/renilla luciferase is displayed. Error bar indicates standard error of the mean (*n* = 2 technical repeats). Similar results have been obtained in two independent biological repeats. **e** SZCs were infected with lentivirus encoding only dCas9-KRAB or with lentivirus programmed to encode both dCas9-KRAB plus guide RNAs targeting the various SZC-enriched ATAC-seq peaks (two to three different guides for each ATAC-Seq peak) that surround the *Prg4* locus. After selection in puromycin, the cells were cultured in the presence of TGF-β2 (20 ng/ml) for 3 days. Gene expression was assayed by RT-qPCR and normalized to *Creb5*. Relative levels of *Prg4* expression in cells expressing dCas9-KRAB plus various guide RNAs (lanes 2–10) are compared to cells expressing only dCas9-KRAB (lane 1). Error bar indicates standard error of the mean (*n* = 2 technical repeats). Similar results have been obtained in three independent biological repeats. **f** Model for Creb5-dependent induction of *Prg4* expression.

Because the above findings collectively implicate Creb5 as a key regulator of *Prg4* expression, we sought to understand the basis for its crucial role in driving SZC-specific gene expression. Active enhancers and promoters are both marked by relatively accessible regions of chromatin (reviewed in^50^). To identify genomic sites with differential chromatin access in SZCs and DZCs, we performed the assay for transposase-accessible chromatin (ATAC-seq,^51^) on nuclei isolated separately from these populations of primary bovine articular chondrocytes. The 907 sites selectively accessible in SZCs were enriched for five distinct sequence motifs, including CRE (cAMP response elements; TGACGTCA), TRE (TPA response elements; TGAGTCA), Nfi, Nfat, and Tbx binding motifs (Fig. 6a). Notably, both CRE and TRE motifs bind Creb5, either as a homodimer or as a heterodimer with Jun^40^. In contrast, ATAC-Seq peaks that were selectively accessible in DZCs were most highly enriched for two distinct sequence motifs, which serve as binding sites for either Tead or Runx TFs (Fig. 6b). Notably, among genes enriched in either SZCs or DZCs which contained transcription start sites located ≤25 kb from differentially accessible ATAC sites, we observed a high correlation between chromatin access and zone-specific gene expression (Fig. 6c). This correlation was highest at the *Prg4* locus (Fig. 6c), where four distinct regions (E1–E4) were selectively accessible in SZCs (Fig. 7a). Chromatin IP (ChIP) of iCreb5-DZCs with HA antibody, followed by PCR analysis of the precipitated DNA, revealed specific occupancy of Creb5 at the two *Prg4* promoter-proximal sites, E1 and E2, and increased binding induced by TGF-β treatment at both candidate *cis*-elements (Fig. 7b). Together, these observations implicate CRE/TRE binding TFs such as Creb5 in distinguishing the two articular chondrocyte populations. Atf2, which is closely related to Creb5, has been found to directly interact with Smad3/4^52^. We observed that Creb5 can similarly co-immunoprecipitate Smad2/3 (Supplementary Fig. 4), suggesting that TGF-β signaling may in part increase *Prg4* expression by inducing nuclear translocation of Smad2/3 and consequent interaction with Creb5.

As *Prg4* is a seminal SZC-specific marker, we examined whether SZC-specific ATAC-Seq peaks E1–E4 function as enhancers. To this end, we appended each sequence upstream of the *Prg4* promoter and firefly luciferase cDNA; and assessed the constructs’ ability to drive reporter gene expression in primary bovine SZCs or DZCs. While the *Prg4* promoter alone showed little activity, addition of both E1 and E2 upstream elements drove luciferase expression specifically in SZCs, but TGF-β2 did not add to this effect (Fig. 7c). Thus, E1 and E2 both interact with Creb5-HA (in iCreb5-DZCs) and drive SZC-specific gene activity, but do not respond to TGF-β. In striking contrast, addition of the distal regulatory element E3 to the promoter-proximal E1 and E2 elements robustly induced TGF-β2 responsive luciferase expression, in both SZCs and DZCs (Fig. 7c). The E4 element alone failed to confer a TGF-β response and did not interfere with the potent activity of E3 (Fig. 7c). Lastly, while treatment of iCreb5-DZCs with doxycycline plus TGF-β2 weakly induced expression of the E2E1-Prg4-luciferase construct, the addition of the E3 element to this reporter strongly enhanced doxycycline-dependent luciferase activity (Fig. 7d). Thus, the E3 element, located 140 kb upstream of the *Prg4* transcription start site, contains an enhancer that responds robustly to TGF-β2 in both SZ and DZ chondrocytes.

To ask whether the SZC-enriched ATAC-Seq peaks E1–E4 are necessary to drive *Prg4* expression in SZCs, we infected these cells with a lentivirus encoding either only a dead-Cas9–KRAB fusion protein (dCas9-KRAB) or dCas9-KRAB plus guide RNAs targeting each *cis*-element. Targeting of dCas9-KRAB to putative enhancers recruits the H3K9 methyltransferase SETDB1, hence increasing local H3K9me3 levels and repressing target genes^53^. Compared to dCas9-KRAB alone, inclusion of guide RNAs against E1, E2, E3, or E4 each repressed TGF-β2 induction of *Prg4* (Fig. 7e). Notably, targeting of E1 with two different gRNAs repressed *Prg4* induction to 0.1% of control levels, while targeting of E2, E3, or E4 repressed *Prg4* to 4–36% of control levels (Fig. 7e). While E1–E3 all lie upstream of the *Prg4* coding region, E4 is located in an intron of the *Prg4* gene. Thus, it is possible that recruitment of dCas9-KRAB to E4 and consequent deposition of H3K9me3 modification to the *Prg4* gene proper may block progression of RNA polymerase II, and thereby decrease production of *Prg4* mRNA. Taken together, these findings reveal that expression of *Prg4* in SZCs is dependent upon both the proximal *cis*-elements E1 and E2, which bind Creb5, and a distant E3 element, that drives TGF-β responsive gene activity.

## Discussion

In this work, we document that Creb5 is specifically expressed in the superficial zone of the articular cartilage and is necessary for TGF-β signaling to promote *Prg4* expression in superficial zone bovine articular chondrocytes. In developing mouse knees, Creb5 is expressed in the articular perichondrium, in superficial cells of the prospective meniscus, and in synovial fibroblasts that line the joint cavity; the same tissues that express *Prg4*^2,4,44^. Thus, Creb5 is present in the tissues that express *Prg4* in murine, bovine, and human joints, and is necessary to maintain competence for *Prg4* expression in superficial zone articular chondrocytes. In addition, when misexpressed in deep zone bovine articular chondrocytes, Creb5 confers the competence for TGF-β and EGFR signals to induce *Prg4* expression in these cells. The regionalized expression of Creb5 in the articular cartilage, which is confined to the superficial zone, helps to explain how *Prg4* expression is similarly constrained to this region of the articular cartilage. As nuclear-localized CREB5 is specifically present in the lubricin-expressing superficial zone of articular cartilage in adult human femoral head articular cartilage, it is plausible that this TF may also be necessary to sustain robust lubricin expression in adult human articular cartilage. Loss of Creb5 function in SZCs decreased the expression of both *Prg4* and other SZC-specific genes (such as *Epha3*). Thus, it seems likely that *Creb5* may play a larger role in maintaining the unique biological properties of the superficial zone of the articular cartilage.

Prior work has indicated that mechanical motion can promote the expression of *Prg4* in articular cartilage via multiple *Creb1* dependent, fluid flow shear stress-induced signaling pathways^33^. In contrast to *Creb5*, whose expression is restricted to the superficial zone of the articular cartilage, *Creb1* transcripts are equally expressed in both superficial and deep zones of this tissue; but at a lower level than *Creb5*. While both *Creb1* and *Creb5* can bind to overlapping binding sites^40,54^, their transcriptional activities are modulated by distinct signaling pathways. PKA-mediated phosphorylation of Creb1 (on Ser133) promotes the interaction of this TF with the KIX domain of the co-activator proteins CBP (CREB-binding protein) and/or p300^55–57^. Both PGE2 and PTHrP signaling pathways (or forskolin treatment), which can all activate PKA, promote *Prg4* expression in cultures of epiphyseal chondrocytes taken from 5-day-old mice^33^. Despite its name, *Creb5* is most closely related to the ATF2/7 family^40^, whose transcriptional activity is regulated by SAPK and MAPK signaling pathways (reviewed in^42,43^). Interestingly, the PKA activator 8-Br-cAMP can induce phosphorylation of ATF2 on T71^58^. As this phosphorylation site is conserved in Creb5, it is possible that PKA signaling may similarly be able to regulate Creb5 activity. Future ChIP-Seq analysis will be necessary to evaluate whether Creb1 and Creb5 share overlapping binding sites on *Prg4* regulatory elements, and whether these TFs work either synergistically or in parallel to activate the expression of *Prg4* in response to differing signaling pathways.

By performing both ATAC-Seq and ChIP for Creb5, we have found that Creb5 directly binds to two *Prg4* promoter-proximal regulatory elements (E1 and E2), which display an open chromatin conformation specifically in superficial zone articular chondrocytes. Interestingly, the *Prg4* promoter-proximal regulatory elements (E1 and E2), which interact with Creb5, can drive superficial zone-specific chondrocyte gene expression, but cannot respond to TGF-β signals. In striking contrast, appending a distal 5′ regulatory element (E3), which also displays increased chromatin accessibility in superficial zone articular chondrocytes (but does not directly bind to Creb5), immediately adjacent to the more proximal Creb5 binding elements (in a luciferase reporter construct) drove robust luciferase expression in response to TGF-β2. Recruitment of a dCas9-KRAB fusion protein to either the Prg4 promoter-proximal regulatory elements (that bind to Creb5) or to more distal regulatory elements blunted induction of *Prg4* by TGF-β signals in superficial zone chondrocytes. Our working hypothesis is that direct interaction of Creb5 with either E1 or E2 alters the chromatin structure of these regulatory elements, such that they can interact with more distal regulatory elements, which, in turn, drive robust TGF-β-dependent induction of *Prg4* (Fig. 7f). It may be relevant in this regard that Creb5 can bind to DNA as a heterodimer with Jun; and that Jun/Fos (in an AP1 complex) can recruit the SWI/SNF (BAF) chromatin remodeling complex to establish accessible chromatin on targeted enhancer elements^59^. Future studies will be necessary to investigate whether Creb5 can similarly remodel chromatin to establish enhancer accessibility for the interaction of other TFs.

Consistent with the restricted expression of Creb5 in the superficial zone of the articular cartilage, we observed that Creb5 binding sites^40^, including CRE (cAMP response elements; TGACGTCA) and TRE (TPA response elements; TGAGTCA), were both enriched in SZC-specific ATAC-Seq peaks. In contrast, binding sites for either Tead or Runx TFs were enriched in DZC-specific ATAC-Seq peaks. While it is currently unclear what regulates either the SZC-specific expression of *Creb5* or the occupancy of Tead and Runx TFs on DZC-specific accessible regions of chromatin, these findings underscore the differential transcriptional regulation in these two regions of the articular cartilage. Notably, *Prg4*-expressing cells in embryonic and neonatal mice have been found to give rise to all regions of the articular cartilage in adult animals, including those in the deep zone of this tissue^8–10^. Thus, the absence of *Creb5* expression in deep zone articular chondrocytes suggests that *Creb5*, which is initially expressed in the precursors of all articular chondrocytes, is somehow downregulated together with *Prg4*/lubricin in the deep zone of the articular cartilage.

TGF-β^36,37^, EGFR^38^, and Wnt/β-catenin^11,34,35^ signaling have all been shown to be necessary to maintain the expression of *Prg4* in articular cartilage. Interestingly, we observed that TGF-β and EGFR signaling pathways augment the ability of either endogenous Creb5 or exogenous iCreb5 to promote expression of *Prg4* in either superficial or deep zone bovine articular chondrocytes, respectively. Binding of Creb5 to *Prg4* promoter-proximal regulatory elements (E1 and E2) was considerably enhanced by TGF-β administration, consistent with our finding that Creb5, like ATF2^52^, can be co-immunoprecipitated with Smad2/3. Thus, TGF-β signaling may increase occupancy of Creb5 on the two *Prg4* promoter-proximal enhancer elements (E1 and E2) by inducing nuclear translocation of Smad2/3 and consequent interaction with Creb5. Future studies will be necessary to determine whether the requirement for Wnt^11,34,35^ and EGFR^38^ signals to maintain the expression of *Prg4* in articular cartilage is due to modulation of either the expression or activity of Creb5, or of other necessary co-factors. Exogenous Creb5 could drive relatively high-level expression of *Prg4* (approximately equal to 75% of *Gapdh* transcript levels) in deep zone bovine articular chondrocytes treated with TGF-β. In contrast, the combination of *Creb5* and TGF-β signaling only induced *PRG4* expression to ~0.2% of *GAPDH* transcript levels in either a human chondrosarcoma cell line (SW1353) or in an immortalized human costal chondrocyte cell line (C-28/12)^49^. Thus, it seems likely that in addition to Creb5, other transcriptional regulators that are unique to articular chondrocytes may play a role in driving high-level level expression of *Prg4*. In addition to Creb5, we found that 28 transcriptional regulators were more highly expressed (at least twofold) in SZCs than in DCZs; and only one TF (Sall1) was more highly expressed in DZCs (Table 1). Notably, in addition to TRE and CRE sites (which are recognized by Creb5^40^), Nfi, Nfat, and Tbx binding motifs were significantly enriched in SZC-specific ATAC-Seq peaks (Fig. 6a), suggesting that members of the Nfi, Nfat, and Tbx TF families may cooperate with Creb5 to promote SZC-specific gene expression. Indeed, cartilage-specific deletion of *Nfatc1* in *Nfatc2-*null mice leads to early onset OA, and decreased expression of *Prg4*^32^. Future studies will be necessary to determine whether Nfi, Nfat, or Tbx TFs (or any of the other SZC-enriched transcriptional regulators) play a direct role in driving the expression of either *Prg4* or other SZC-specific genes.

Creb5 shares a high degree of homology with both Atf2 and Atf7 in both its DNA-binding domain and its N-terminus, which contains two highly conserved Threonine-Proline sequences (T59 and T61 in Creb5) that are substrates for p38 kinase, JNK, and ERK (reviewed in^42,43^). We have found that phosphorylation of Creb5 (T61) can be boosted by both EGFR and TGF-β signals in superficial zone chondrocytes and that phosphorylation of Creb5 (T61) is blocked in TGF-β treated iCreb5-DZCs by either a p38 inhibitor (SB203580) or a JNK inhibitor (SP600125). In addition, we noted that SAPK function is required to promote *Prg4* expression in either superficial zone chondrocytes (that express endogenous Creb5) or iCreb5-DZCs (programmed to express iCreb5). Substitution of alanine for threonine in the two highly conserved SAPK phosphorylation sites of either Atf2 or Atf7 cripples the activity of the adjacent N-terminal transcriptional activation domain of these proteins^60,61^ and the biological activity of Atf2 in vivo^62^. In striking contrast, we found that similar mutations in the SAPK phosphorylation sites of Creb5 (i.e., iCreb5-T59,61A) did not depress the ability of this TF to induce *Prg4* expression in deep zone chondrocytes treated with either an EGFR ligand and/or TGF-β signals. These findings indicate that while SAPKs can promote phosphorylation of Creb5 (on T59, T61), induction of *Prg4* expression, by EGFR and TGF-β signals, is dependent upon SAPK-mediated phosphorylation of either other sites in Creb5 or phosphorylation of other substrates. The transcriptional activity of a chimeric protein containing the GAL4 DNA-binding domain fused to the N-terminus of Creb5 (GAL4-Creb5 (1-128)) was attenuated by simultaneous T59A/T61A mutations. Thus, it will be interesting to determine whether, in contrast to *Prg4*, the induction of other Creb5 target genes are regulated by phosphorylation of T59 and T61 in Creb5.

## Methods

### Vertebrate animals

All work with vertebrate animals was approved by the Harvard Medical School Institutional Animal Care and Use Committee (IACUC).

### Isolation and culture of bovine articular chondrocytes

The knee joints from 1- to 2-week-old bovine calves were obtained from Research 87 (a local abattoir in Boylston, MA) directly after slaughter. The intact femoropatellar joints were isolated by transecting the femur and mounting the distal segment in a drilling apparatus. The femoropatellar articular cartilage was then exposed by opening the joint capsule, severing the medial, lateral, and cruciate ligaments, and removing the tibia, patella, and surrounding tissue. Four to six cylindrical cores of cartilage and underlying bone, 9.5 mm in diameter and −15 mm deep, were drilled from each facet (medial and lateral) of the femoropatellar groove. During this entire process, the cartilage was kept moist and free of blood by frequent rinsing with sterile PBS supplemented with antibiotics (100 U/ml penicillin and 100 μg/ml streptomycin). Each core was then inserted into a cylindrical sample holder for a sledge microtome (Model 860, American Optical, Buffalo, NY). An initial ~200–300 micron thick slice of superficial zone articular cartilage (SZC) was first harvested via the microtome. The next ~4 mm thick slice of middle zone cartilage tissue was then removed from the same core and discarded. Finally, an 800 micron to 1 mm thick slice of deep zone articular cartilage (DZC) was then harvested from the same core. All of the superficial zone slices from a given knee joint were placed in a 50 mL centrifuge tube filled with medium (DMEM with 10 mM HEPES, 0.1 mM nonessential amino acids, and additional 0.4 mM proline, 25 pg/ml ascorbate) and supplemented with 10% fetal bovine serum. Similarly, all deep zone slices from a given joint were placed in a separate tube filled with medium and serum. To isolate chondrocytes from the superficial and deep zone slices, deep zone cartilage shavings were chopped into pieces about 1 mm^3^. There was no need to chop superficial zone shavings as they are thin enough. Both superficial zone and chopped deep zone cartilage were digested in 10 ml of pronase (1 mg/ml) in DMEM with 1% penicillin and streptomycin for 1 h. Pronase was replaced by collagenase D (1 mg/ml) in DMEM with 1% penicillin and streptomycin and cartilage tissue was digested in the incubator at 37 degrees overnight. The next day, the dissociated cells were filtered through a 70 μm strainer, counted, pelleted for 5 min at 1200 rpm, and resuspended in DMEM/F12 plus 10% FBS. For lentivirus infection the cells were plated into a six-well plate (approximately 1 million cells per well). Twenty-four hours after plating, the medium was changed with new DMEM/F12 plus 10% FBS.

### RNA-Seq analysis

Newly isolated bovine superficial zone chondrocytes and deep zone chondrocytes were cultured (in DMEM/F12 plus 10% FBS) for 3 days prior to performing RNA-Seq analysis. RNA from superficial zone chondrocytes and deep zone chondrocytes were purified using Trizol reagent (Life Technologies). Genomic DNA in RNA samples was removed using TURBO DNA-free™ Kit (Thermo Fisher Scientific, Cat#: AM1907). Total RNA (5–10 ng) was purified using the manufacturer’s instructions and used to prepare libraries with SMART-Seq v4 Ultra-Low Input RNA Kit (Clontech) followed by sequencing on a NextSeq 500 instrument (Illumina) to obtain 75-bp single-end reads. Raw RNA-seq reads were assessed with FastQC 0.11.3 followed by MultiQC 1.2 aggregation^63^ to determine sequence quality, per-base sequence quality, per-read GC content (~50), and per-base N content. Read pairs were aligned to the Bos taurus genome Ensembl build UMD 3.1 version 88 using STAR 2.5.2b^64^ employing a custom index, with read counting for an unstranded library preparation. Counts were normalized using Trimmed Means of *M* values^65^ as part of the edgeR package^66,67^, and modeled for biological and gene-wise variation. Differential expression between sample types was determined through the Exact Test in edgeR, with Benjamini–Hochberg^68^ multiple testing correction (FDR) set at < 0.05.

### ATAC-Seq analysis

Newly isolated bovine superficial zone articular chondrocytes and deep zone articular chondrocytes were cultured (in DMEM/F12 plus 10% FBS) for 3 days prior to performing ATAC-Seq analysis. ATAC-Seq^51,69^ was performed on duplicate samples of 8000–35,000 superficial zone chondrocytes or deep zone chondrocytes. Cultured chondrocytes were first digested into single cells using trypsin, and then trypsin was neutralized by serum. Digested single cells were washed twice in ice-cold PBS, resuspended in 50 μl ice-cold ATAC lysis buffer (10 mM Tris·Cl, pH 7.4, 10 mM NaCl, 3 mM MgCl_2_, 0.1% (V/V) Igepal CA-630), and centrifuged at 500 × *g* at 4 °C to isolate nuclear pellets. Nuclear pellets were treated with Nextera Tn5 Transposase (Illumina, FC-121-1030) in a 50 μl reaction for 30 min at 37 °C. Transposed DNA was immediately isolated using a Qiagen MinElute PCR purification kit, and then PCR amplified in a 50 μl reaction using a common forward primer and different reverse primers with unique barcodes for each sample as per^69^. After five cycles of PCR, 45 μl of the reaction was kept on ice; while 5 μl reaction was amplified by RT-qPCR for 20 cycles to determine the cycles required to achieve 1/3 of the maximal RT-qPCR fluorescence intensity. The remaining 45 μl of the reaction was then amplified by eight additional cycles to achieve 1/3 of the maximal RT-qPCR fluorescence intensity (as determined above). The amplified DNA was purified using a Qiagen MinElute PCR purification kit and primer dimers (<100 bp) were removed using AMPure beads (Beckman Coulter). ATAC-Seq was performed with two biological repeats to ensure the robustness of the data sets. Raw ATAC-Seq reads were aligned to the bovine genome (Bostau 6) using Bowtie2^70^. Aligned signals in raw (bam) files were filtered to remove PCR duplicates and reads that aligned to multiple locations. Peaks were identified using MACS v1.4^71^.

### Construction of lentivirus encoding shRNA targeting Creb5

Bovine Creb5 shRNAs targeting the Creb5 3′UTR were designed using Block-iT RNAi designer (Thermo Fisher). The most efficient Creb5 shRNA sequence we identified was: CCG GGC CTT CAA GAA GAG CTG TTG CCT CGA GGC AAC AGC TCT TCT TGA AGG CTT TTT G (targeting sequence is underlined; employed in Fig. 3a–c). Oligos (ordered from Integrated DNA Technologies) were annealed in a 10 μl reaction (1 μl Forward oligo (100 μM), 1 μl Reverse oligo (100 μM), 1 μl T4 ligation buffer (10X), 6.5 μl nuclease-free H_2_O, 0.5 μl T4 polynucleotide kinase (NEB)) in a PCR machine, programmed to cycle: 37 °C 30 min, 95 °C 5 min, and then ramp down to 25 °C at 5 °C/min. Annealed oligos that are compatible with the sticky ends of EcoRI and AgeI were diluted (1:100) and cloned into pLKO.1 TRC-Cloning vector (Addgene # 10878) that had been digested with EcoRI and AgeI, and gel purified. Lentiviral vector encoding shRNA targeting bovine Creb5 (shCreb5) was verified by sequencing (Genewiz). Lentiviral control vector containing a scrambled shRNA (shSCR) was ordered from Addgene (scramble shRNA, Addgene # 1864).

### CRISPR/Cas9 targeting the DNA-binding domain of *Creb5*

The CRISPR/Cas9 system was used to introduce insertions/deletions (indels) into the *Creb5* DNA-binding domain in bovine superficial zone chondrocytes. Briefly, sequence-specific sgRNAs that guide Cas9 to the genomic region encoding the *Creb5* DNA-binding domain were designed following the instructions located at (http://crispr.mit.edu/). The most efficient guide targeting the DNA-binding domain of bovine *Creb5* that we identified is: C TGA AGC TGC ATG TTT GTC T (employed in Fig. 3d–f and Supplementary Fig. 1). Oligos (ordered from Integrated DNA Technologies) were annealed in a 10 μl reaction (1 μl forward oligo (100 μM), 1 μl reverse oligo (100 μM), 1 μl T4 ligation buffer (10X), 6.5 μl nuclease-free H_2_O, 0.5 μl T4 polynucleotide kinase (NEB)) in a PCR machine programmed to cycle: 37 °C 30 min, 95 °C 5 min, and then ramp down to 25 °C at 5 °C/min. The annealed oligos were diluted (1:200) and cloned into lentiCRISPRv2 (Addgene # 52961) using a Golden Gate Assembly strategy (containing: 100 ng circular lentiCRISPRv2, 1 μl diluted oligo, 0.5 μl BsmBI (Thermo Fisher), 1 μl Tango buffer (10X), 0.5 μl DTT (10 mM), 0.5 μl ATP (10 mM), 0.5 μl T4 DNA Ligase (NEB), H_2_O up to 10 μl) with 20 cycles of: 37 °C 5 min, 21 °C 5 min. The ligation reaction was then treated with PlasmidSafe (Epicentre, Cat#: E3101K) to digest any residual linearized DNA. PlasmidSafe treated plasmid was transformed into Stbl3 competent cells (Thermo Fisher). LentiCRISPRCreb5gRNA plasmid containing the Creb5 gRNA was verified by sequencing (Genewiz). Either the parental vector, lentiCRISPRv2, or lentiCRISPRv2CTRgRNA (Addgene # 107402) was used to generate control lentivirus.

### T7 endonuclease I (T7E1) assay to detect indels in *Creb5*

The T7E1 assay was used to detect on-target CRISPR/Cas9 induced insertions and deletions (indels) in cultured cells. cDNA derived from mRNA isolated from either lentiCRISPRv2 or lentiCRISPRCreb5gRNA infected bovine superficial zone chondrocytes was employed for the T7E1 assay. A 210 bp cDNA fragment, which flanks the Creb5 gRNA cleavage site, was amplified using Creb5 RT-qPCR primers (Supplementary Table 1; Creb5 primers 1) in a 25 μl PCR reaction (12.5 μl Q5 High-Fidelity 2X Master Mix, 1.25 μl Forward Primer, 1.25 μl Reverse Primer, cDNA, nuclease-free H_2_O to a final volume of 25 μl). After denaturation of the cDNA at 98 °C 30 s; the PCR machine was programed to cycle 35 times at: 98 °C 10 s, 60 °C 15 s, 72 °C 15 s; 72 °C 2 min; 4 °C hold. The PCR product was denatured and annealed in an 18 μl reaction (15 μl PCR product, 2 μl NEB Buffer2 (10X), 1 μl nuclease-free H_2_O) with the following the cycling conditions: 95 °C 10 min; 95–85 °C (ramp rate of −2 °C/s); 85–25 °C (ramp rate of −2 °C/s). After denaturing and reannealing the PCR products, T7E1 (2 μl) was added, and the mixture was incubated at 37 °C for 60 min. Cleavage of the PCR products by T7 endonuclease was assayed by agarose gel electrophoresis.

### Generation of lentivirus encoding either WT or mutant iCreb5

Total RNA (containing *Creb5* transcripts) was isolated from bovine superficial zone articular chondrocytes using Trizol reagent (Life Technologies). cDNA was reverse transcribed using the oligo dT reverse transcription kit SuperScript® III First-Strand Synthesis System (Life Technologies, Cat. No. 18080051). The bovine *Creb5* open reading frame (508aa; see NM_001319882.1) was predicted by RNA-Seq of bovine superficial zone articular chondrocytes. A 1527 bp cDNA fragment (encoding 508aa plus the stop codon) was PCR amplified from bovine superficial zone articular chondrocyte cDNA using the Q5 High-Fidelity 2X Master Mix (NEB, Cat. No. M0492S) (forward primer: AT GAT TTA TGA GGA ATC C; and reverse primer: TTA GAG GAT GGG GTT CAG GT) and then cloned into the pCR®-Blunt vector (Life Technologies, Cat#: K270020). Using the pCR®-Blunt-bovine Creb5 as a template, a fragment of DNA (TAC CCT TAC GAC GTC CCA GAC TAC GCT GGC TCC TAC CCT TAC GAC GTC CCA GAC TAC GCT TAC CCT TAC GAC GTC CCA GAC TAC GCT) encoding 3xHA tags was added onto the C-terminus of Creb5 (immediately before the stop codon). HA-tagged Creb5 was then cloned into a Gateway vector (pENTR-Creb5-HA) using the pENTR™/SD/D-TOPO® Cloning Kit (Life Technologies, Cat#: K2420-20). Creb5-HA was then transferred from pENTR-Creb5-HA into the pInducer20 (Addgene # 44012) lentivirus destination vector or into the pLenti CMV Puro DEST (w118-1) vector (Addgene Plasmid #17452) using Gateway LR Clonase (Thermo Fisher, Cat. No. 11791020), to generate pInducer20-iCreb-WT or pLenti-Creb5, respectively. pLenti-GFP vector (EX-EGFP-Lv102) was purchased from GeneCopoeia. To generate lentivirus vectors encoding iCreb5 mutants, the DNA sequence encoding T59/T61 sites in pENTR-Creb5-HA was mutated into sequence encoding either T59/T61A or into T59/T61D, respectively, using the Q5® Site-Directed Mutagenesis Kit (NEB, Cat#: E0554S) following the manufacturer’s instructions. Creb5 mutants were then cloned into the lentivirus destination vector pInducer20 using Gateway technology.

### Lentivirus encoding dCas9-KRAB targeting *Prg4* ATAC-seq peaks

The CRISPR interference system was used to study the function of *Prg4* enhancer elements (E1–E4) in bovine superficial zone chondrocytes. Briefly, sequence-specific sgRNAs that direct a dCas9-KRAB fusion protein (dCas9-KRAB;^53^) to the genomic region of the *Prg4* enhancer elements were designed following the instructions at CHOPCHOP (http://chopchop.cbu.uib.no/). Oligos (ordered from Integrated DNA Technologies) were annealed in a 10 μl reaction (1 μl forward oligo (100 μM), 1 μl reverse oligo (100 μM), 1 μl T4 ligation buffer (10X), 6.5 μl nuclease-free H_2_O, 0.5 μl T4 polynucleotide kinase) in a PCR machine programmed to cycle: 37 °C 30 min, 95 °C 5 min and then ramp down to 25 °C at 5 °C/min. The annealed oligos were diluted (1:200) and cloned into pLV hU6-sgRNA hUbC-dCas9-KRAB-T2a-Puro (encoding dCas9-KRAB; Addgene # 71236) using a Golden Gate Assembly strategy (containing: 250 ng circular pLV hU6-sgRNA hUbC-dCas9-KRAB-T2a-Puro, 1 μl diluted oligo, 0.5 μl BsmBI (Thermo Fisher), 1 μl Tango buffer (10X), 0.5 μl DTT (10 mM), 0.5 μl ATP (10 mM), 0.5 μl T4 DNA Ligase (NEB), H2O up to 10 μl) with 20 cycles of: 37 °C 5 min, 21 °C 5 min. The ligation reaction above was then treated with PlasmidSafe (Epicentre, Cat#: E3101K) to digest any residual linearized DNA. PlasmidSafe treated plasmid was transformed into Stbl3 (Thermo Fisher) competent cells. dCas9-KRAB plasmids containing the various Prg4 enhancer gRNAs were verified by sequencing (Genewiz). The gRNAs targeting the bovine Prg4 enhancer elements are listed in Supplementary Table 4.

### Growth and purification of lentivirus

HEK293 cells were used to package lentivirus. HEK293 cells were plated in a 15-cm dish in 25 ml of DMEM/F12 (Invitrogen) supplemented with 10% heat-inactivated fetal bovine serum (Invitrogen) and Pen/Strep at 37 °C with 5% CO_2_. Transfection was performed when the cells were ~70–80% confluent. Lentiviral expression plasmid (6 μg), psPAX2 (4.5 μg, Addgene #12260), and pMD2.G VSVG (1.5 μg, Addgene #12259) plasmids were added into a sterile tube containing 500 μl of Opti-MEM® I (Invitrogen). In a separate tube, 36 μl of Fugene 6 was diluted into 500 μl of Opti-MEM I. The diluted Fugene 6 reagent was added dropwise to the tube containing the DNA solution. The mixture was incubated for 15–25 min at room temperature to allow the DNA-Fugene 6 complex to form. The DNA-Fugene 6 complex was directly added to each tissue culture dish of HEK293 cells. After cells were cultured in a CO2 incubator at 37 °C for 12–24 h, the medium (containing the DNA-Fugene 6 complex) was replaced with 36 ml fresh DMEM/F12 medium supplemented with 10% heat-inactivated fetal bovine serum and penicillin–streptomycin. Cells were again placed in the CO2 incubator at 37 °C; and virus-containing culture medium was collected in sterile capped tubes 48, 72, and 96 h post transfection. Cell debris was removed by centrifugation at 500 × *g* for 10 min and filtration through nylon low protein-binding filters (SLHP033RS, Millipore). Virus was concentrated by ultracentrifugation (employing a SW32 rotor at 25 K for 2 h and 30 min at 4 °C) and stored at −80 °C.

### Infection of chondrocytes with lentivirus and RT-qPCR

Newly isolated bovine superficial zone articular chondrocytes, deep zone articular chondrocytes, SW1353 cells, or immortalized human costal chondrocyte cells (C-28/I2;^49^) were cultured for at least 2–3 days before infection. SW1353 cells were obtained from ATCC (HTB 94). The immortalized human costal chondrocyte cell line (C-28/I2)^49^ was obtained from Dr. Mary Goldring (Hospital for Special Surgery, Weill Cornell Medical College & Weill Cornell Graduate School of Medical Sciences). Ultracentrifuge-concentrated lentivirus was added into DMEM/F12 medium (with 10% FBS) containing 7.5 μg/ml DEAE-Dextran (to increase infection efficiency). Twenty-four hours after infection, medium was replaced with new DMEM/F12 medium (with 10% FBS) containing either 0.8 μg/ml puromycin (for 5 days) or 500 μg/ml G418 (for 11 days) for selection. To increase the efficiency of *Creb5* mutation in cells infected with either LentiCRISPRCreb5gRNA, lentiCRISPRv2, or lentiCRISPRv2CTRgRNA (Addgene #107402), cells were cultured for at least an additional 14 days, prior to assaying gene expression. Cells were replated onto low attachment tissue culture plates (Corning #3471 or #3473) in DMEM/F12 medium (with 10% FBS), without either puromycin or G418. After 2–3 days culture, RNA was harvested using Trizol reagent and cDNA were synthesized using SuperScript™ III First-Strand Synthesis SuperMix (Invitrogen, Cat#: 11752-050) according manufacture’s guidelines. RT-qPCR primers were synthesized by Integrated DNA Technologies. RT-qPCR was performed in an Applied Biosystem 7500 Fast Real-Time PCR Machine. Each experiment was performed with two to three biological repeats and each RT-qPCR assay was performed with technical repeats. Prism statistical software (GraphPad) was employed to analyze the data. In RT-qPCR experiments, gene expression was normalized to that of either *Gapdh* or *Creb5*, as indicated. Gene expression was analyzed by RT-qPCR, employing at least one primer which was composed of sequences encoded by distinct exons. All RT-qPCR primers are listed in Supplementary Tables 1 and 10. As indicated in Supplementary Table 1, Creb5 (Primers 1), which amplify the coding region of bovine Creb5 cDNA, were used to detect both endogenous Creb5 and exogenous iCreb5 transcripts. Creb5 (Primers 2), which amplify sequences that are unique to the Creb5 3′UTR (and are absent from iCreb5), were used to specifically detect endogenous Creb5 expression.

### Construction of GAL4-Creb5 fusion constructs

Using the pCR®-Blunt-bovine Creb5 cDNA as the template, Creb5 (1-128) and Creb5 (1-508) fragments were PCR amplified to lie between a 5′ EcoR1 and 3′ Xba1 site, using Q5 High-Fidelity 2X Master Mix (NEB, Cat#: M0492S). These amplicons were then cloned into EcoR1/Xba1 cleaved Gal4-HA-HIF1α (530-652) plasmid (Addgene # 24887), following the removal of the EcoRI-HA-HIF1α-XbaI fragment from this plasmid. The resulting plasmids were GAL4-Creb5 (1-128) and GAL4-Creb5 (1-508). GAL4-Creb5 (1-128) T59/T61A and GAL4-Creb5 (1-128) C18/C23S were generated by inducing point mutations in the GAL4-Creb5 (1-128) vector using the Q5® Site-Directed Mutagenesis Kit (NEB, Cat#: E0554S). The control vector GAL4 (1-147) was generated by introducing a stop codon immediately after the GAL4 (1-147) sequence in the GAL4-Creb5 (1-128) vector. All the plasmids were verified by sequencing.

### *Prg4-*luciferase constructs and luciferase assays

*Prg4* enhancer/promoter-firefly luciferase constructs were constructed by cloning various *Prg4* regulatory fragments into pGL4.10[*luc2*] vector (Promega). Bovine genomic DNA was extracted from bovine chondrocytes and was used as the template to amplify *Prg4* regulatory regions. A 543 bp fragment encoding the *Prg4* promoter and 5′UTR up to the start codon of *Prg4* was amplified (with flanking BglII-HindIII sites) and cloned into pGL4.10[*luc2*] to generate pGL4.10 *Prg4* promoter-luciferase. A 1061 bp fragment encoding *Prg4* enhancer 1, the *Prg4* promoter, and the 5′UTR up to the start codon of *Prg4* was amplified (with flanking BglII-HindIII sites) and cloned into pGL4.10[*luc2*] to generate pGL4.10 E1-*Prg4* promoter-luciferase. A 585 bp *Prg4* Enhancer2 fragment was amplified (with flanking EcoRV-BglII sites) and cloned into pGL4.10 E1-*Prg4* promoter-luciferase to generate pGL4.10 E2E1-*Prg4* promoter-luciferase. A 802 bp *Prg4* Enhancer3 fragment was amplified (with flanking XhoI-EcoRV sites) and cloned into pGL4.10 E2E1-*Prg4* promoter-luciferase to generate pGL4.10 E3E2E1-*Prg4* promoter-luciferase. A 331 bp *Prg4* Enhancer4 fragment was amplified (with flanking NheI-XhoI sites) and cloned into pGL4.10 E3E2E1-*Prg4* promoter-luciferase to generate pGL4.10 E4E3E2E1-*Prg4* promoter-luciferase. High-fidelity DNA polymerase (NEB, Cat. No. M0492S) was used to generate all the constructs. All the plasmids were verified by sequencing. Prg4 promoter and enhancer 1–4 sequences are listed in Supplementary Table 3.

*Prg4* enhancer/promoter-firefly luciferase reporters were co-transfected together with the pGL4.75 [hRlu/CMV] renilla luciferase reporter (Promega) into either superficial zone chondrocytes, deep zone chondrocytes, or deep zone chondrocytes previously infected with iCreb5-WT lentivirus, using Lipofectamine^TM^ LTX (Thermo Fisher, Cat#: 15338030). Twelve hours after transfection, cell culture medium was changed into new medium to remove the Lipofectamine DNA complex. After another 12 h culture, transfected cells was replated into ultra-low attachment tissue culture dishes (Corning, Cat#: 3471) for an additional 48 h culture, in the either the absence or presence of TGF-β and doxycycline. Cells were harvested and luciferase activity was measured using the Dual-Luciferase® Reporter Assay System (Promega, Cat#: E1960) in a Turner Biosystems Modulus Microplate Reader. Each experiment was performed with two to three biological repeats and each luciferase assay was performed with technical repeats.

### Chromatin IP (ChIP)-PCR analysis

Bovine deep zone articular chondrocytes infected with iCreb5-HA lentivirus were cultured in ultra-low attachment tissue culture dishes (Corning, Cat#: 3471) for 2–3 days in DMEM/F12 supplemented with 10% FBS, in either the absence or presence of doxycycline or doxycycline plus TGF-β2. The chondrocytes were gently centrifuged (200 × *g*, 5 min), and digested with 0.25% Trypsin-EDTA (Thermo Fisher, Cat#: 25200056) at 37 °C for 7 min (until the tissue was dispersed into single cells). Trypsinization was stopped by adding 10x volume 10% FBS/DMEM. Digested single cells were fixed in 1% Formaldehyde at room temperature for 20 min with shaking. Formaldehyde was quenched with 125 mM glycine on a shaking platform at room temperature for 10 min. Cells were then washed two times with cold PBS containing protease inhibitor cocktail (Roche) followed by lysis with 500 μl ChIP sonication buffer (0.5% SDS, 10 mM EDTA, 50 mM Tris-HCl pH 8.1). Chromatin (from ~5 million cells in 500 μl ChIP sonication buffer) was sonicated with a Covaris E220 Sonication Machine (PIP = 140, CBP = 200, DF = 5%, Avg Power = 7.0, 300 s each time, six times (Total 30 min)). Debris was removed by spinning samples 8 min at full speed at 4 °C. The chromatin was diluted with ChIP dilution buffer (1.1% TritonX-100, 1.2 mM EDTA, 16.7 mM Tris-HCL pH 8.1, 167 mM NaCl) up to 1500 μl in 1.5 ml eppendorf tube (DNA low bind tube). SDS concentration was adjusted to below 0.2% and TritonX-100 concentration was adjusted to 1%. 5 μg of antibody (against the antigen of choice) was added to the chromatin and immunoprecipitated overnight at 4 °C. Protein A (15 μl) and protein G (15 μl) beads were washed two times with ChIP dilution buffer and then added to the chromatin. The bead-chromatin complex was rotated at 4 °C for 2 h and then washed two times each with low-salt buffer (0.1% SDS, 1% TritonX-100, 2 mM EDTA, 20 mM Tris-HCl pH 8.1, 150 mM NaCl), high-salt buffer (0.1% SDS, 1% TritonX-100, 2 mM EDTA, 20 mM Tris-HCl pH 8.1, 500 mM NaCl), lithium buffer (0.25 M LiCl, 1% IGEPAL CA-630 (or NP-40), 1% deoxycholic acid (sodium salt), 1 mM EDTA, 10 mM Tris pH 8.1), and finally TE buffer (10 mM Tris-HCl pH 8.0, 1 mM EDTA). Chromatin-antibody complexes were eluted twice from beads using 100 μl of elution buffer (1% SDS, 0.1 M NaHCO3) by incubation at room temperature for 10 min. NaCl was added into the 200 μl elute to a final 370 mM concentration. Cross-linked chromatin was reversed at 65 °C with shaking overnight followed by adding RNase A (to a final concentration of 0.2 mg/ml) and incubating at 37 °C for 1 h to digest RNA; and then adding proteinase K (to a final concentration of 0.1 mg/ml) and incubating at 55 °C for 1 h to digest protein. DNA fragments were purified using the PCR purification kit with MinElute Columns (Qiagen) and analyzed using qPCR. In all ChIP-qPCR results, the level of immunoprecipitated DNA was normalized to that of the input DNA. Antibodies employed for ChIP are listed in Supplementary Table 6. Primers employed for ChIP-qPCR are listed in Supplementary Table 2.

### Co-immunoprecipitation

Bovine deep zone articular chondrocytes were infected with a lentivirus encoding doxycycline-inducible Creb5 (3xHA tags were added onto the C-terminus of Creb5). After selection in G418, 6 million cells were cultured in low attachment tissue culture plates (Corning #3262) in DMEM/F12 medium (with 10% FBS, 20 ng/ml TGF-β2) in either the presence or absence of doxycycline for an additional 3 days. Cells were harvested and suspended in 1.5 ml mammalian cell lysis buffer (MLCB: containing 50 mM Tris, pH 7.5, 150 mM NaCl, 0.5 % NP-40,10 mM NaF) with protease inhibitors (PIs) cocktail. The suspension was lysed by gently rocking at 4 °C for 1 h and spun down at max speed on a table top centrifuge for 20 min at 4 °C. In 1.5 ml clear lysate, 100 μl lysate was kept as input and 25 μl of monoclonal anti-HA agarose was added into the remaining 1.4 ml sample. The binding was performed on a rocker at 4 °C overnight. After the binding, the agarose was washed with MLCB plus PIs for at least five times. The precipitated protein was eluted with NuPAGE LDS sample buffer (Invitrogen) plus NuPAGE Sample Reducing Agent (Invitrogen). The anti-HA agarose immunoprecipitated protein was probed with anti-Smad2/3 antibody by western blot. The input protein was probed with anti-Smad2/3 antibody and anti-HA antibody (Supplementary Table 5).

### Western blots

Bovine superficial zone or deep zone articular chondrocytes were collected and lysed in lysis buffer (containing 50 mM Tris. HCl, pH 7.4, 1% NP-40, 0.5% Sodium deoxycholate, 1% SDS, 150 mM NaCl, 2 mM EDTA, 50 mM NaF, PI cocktail (Roche)). The cell lysate were incubated on ice for 15 min, and centrifuged at 3000 × *g*, at 4 °C to remove cell debris. Protein concentration was measured with a Bio-Rad Protein Assay Kit (BIO-RAD, Cat#: 500-0006). The protein was denatured at 70 °C for 10 min with LDS sample buffer (Invitrogen, Cat#: NP0007) and Sample Reducing Agent (Invitrogen, Cat#: NP0009). General SDS-PAGE processing procedures were followed. The blots were visualized by the enhanced chemiluminescence detection method, employing the Pierce ECL Western Blotting Substrate (Pierce, Cat#: 32106). All primary antibodies used for western blots are listed in Supplementary Table 5. Uncropped western blot images are displayed in Supplementary Fig. 5.

### Immunohistochemical analysis

Mouse forelimb, hindlimb, or human articular cartilage (procured by the National Disease Research Interchange) was dissected and fixed in 4% paraformaldehyde (in PBS) at 4 °C overnight, washed with PBS, and incubated in 30% sucrose at 4 °C overnight. Tissues were embedded in OCT and frozen sections were cut at 12 μm using a cryostat. Any nonspecific binding of primary antibodies was blocked by incubation with PBS containing 0.2% Tween 20 and 5% nonimmune goat serum for 1 h at room temperature. Primary antibody incubation was carried out at 4 °C overnight with a rabbit polyclonal antibody to Creb5 (PA5-65593, Thermo Fisher, 1:100), a mouse monoclonal antibody to Prg4 (MABT401, EMD Millipore, 1:50), a rat monoclonal antibody to the HA epitope (11867423001, Sigma, 1:100), or a rabbit polyclonal antibody to Sox9 (AB5535, EMD Millipore, 1:100). After washing in PBST (3 × 15 min at room temperature), sections were incubated with Alexa Fluor 488 or 594 conjugated secondary antibodies (Thermo Fisher, 1:250) for 1 h at room temperature. 4,6-diamidino-2-phenylindole (DAPI; 1 μg/ml) was included with the secondary antibodies to stain DNA. Slides were washed in PBST (3 × 15 min at room temperature) and mounted under a coverslip with Aqua-mount (13800; Lerner Labs). Images were taken at the Nikon Imaging Center at Harvard Medical School. All antibodies employed for immunohistochemistry are listed in Supplementary Table 7.

### In situ hybridization analysis

Digoxigenin (DIG) or fluorescein labeled RNA probes were made using the DIG RNA labeling kit (Roche, Cat. 11175025910) or fluorescein RNA labeling kit (Roche, Cat. No. 11685619910), respectively, per manufacturer’s protocol. T7 RNA polymerase (Roche, Cat. No. 10881767001), T3 RNA polymerase (Roche, Cat. No. 11031171001), or SP6 RNA polymerase (Roche, Cat. No. 10810274001) was employed to generate RNA, depending on the particular RNA probe. After DNase I treatment and precipitation, RNA probes were dissolved in 40 μl RNAse-free water. Forelimbs or hindlimbs were dissected and fixed in 4% paraformaldehyde (made with DEPC-treated PBS) at 4 °C overnight, washed with PBS, and incubated in 30% sucrose (made with DEPC-treated H2O) at 4 °C overnight. Tissues were embedded in OCT and frozen sections were cut at 20 μm using a cryostat. Sections were fixed again with 4% paraformaldehyde (made with DEPC-treated PBS) at room temperature for 5 min, washed twice (5 min each at room temperature) with PBS containing 0.1% Tween 20 (PBST). The sections were digested with proteinase K (4 μg/ml in PBS) at room temperature for 8–20 min (depending on the RNA probe), washed twice with PBST (5 min each), and fixed again with 4% paraformaldehyde (in PBS) at room temperature for 5 min. After washing twice with PBST (5 min each, at room temperature), sections were acetylated for 10 min (at room temperature) in a solution containing 0.25% acetic anhydride and 0.1 M triethanolamine (in DEPC-treated H2O). After washing twice with PBST (5 min each, at room temperature) the slides were rinsed in DEPC-treated H2O, and then air dried. The RNA probe (listed in Supplementary Table 9) was diluted to 100 ng/ml in a 200 μl volume of hybridization solution (as described in^72^), and heated at 95 °C for 5 min before adding to the slide. Hybridization was performed at 65 °C overnight, and washed according to the protocol outlined in^72^. After hybridization, washes, and blocking endogenous peroxidase activity, the slides were blocked with 10% heat-inactivated goat serum at room temperature for 1 h. Anti-DIG-POD antibody (1:100 dilute, anti-DIG-POD, Fab fragment, 11207733910, Roche) or anti-fluorescein-POD antibody (1:100, anti-fluorescein-POD, Fab fragments, 11426346910, Roche) was added and the slides were incubated at 4 °C overnight. After washing in TNT buffer (100 mM Tris-HCl pH 7.5, 150 mM NaCl, 0.05% Tween 20), the biotin (1:100) amplification reagent (TSA Biotin Kit, NEL749A001KT) or fluorescein (1:100) amplification reagent (TSA plus cyanine 3/fluorescein system, NEL753001KT) was applied and slides incubated 20 min at room temperature to develop the signal. Biotin signal was further detected by Streptavidin Secondary Alexa 594 dyes. Slides were washed in TNT buffer (3 × 10 min at room temperature), rinsed in H2O, and mounted under a coverslip with Aqua-mount (13800; Lerner Labs).

### Statistics and reproducibility

RNA-Seq was performed with duplicate biological samples. Differential expression of genes between sample types in these data sets was determined through the Exact Test in edgeR, with Benjamini–Hochberg^68^ multiple testing correction (FDR) set at < 0.05. ATAC-Seq was performed with two biological repeats to ensure the robustness of the data sets. RT-qPCR assays and luciferase assays were performed with two to three independent biological repeats and each assay was performed with two technical repeats.

### Reporting summary

Further information on research design is available in the Nature Research Reporting Summary linked to this article.

## Supplementary information

Supplementary Information

Description of Supplementary Files

Supplementary Data1

Supplementary Data 2

Reporting Summary

## Data Availability

All primary RNA-Seq and ATAC-Seq data sets generated and analyzed during the current study are available in the GEO repository (Accession GSE132379). Primary data for all the graphs and charts in this study are available in Supplementary Data 2. All data and materials used in the analysis will be made available to any researcher for purposes of reproducing or extending the analysis.
