# Supplementary material for: Creb5 establishes the competence for Prg4 expression in articular cartilage
Source: Commun Biol. 2021 Mar 12;4:332. doi: 10.1038/s42003-021-01857-0 (PMC7955038; doi:10.1038/s42003-021-01857-0)
Supplement: Supplementary file 1 — Supplementary Information [file 42003_2021_1857_MOESM1_ESM.pdf]

Supplementary Materials for:

Title: Creb5 establishes the competence for *Prg4* expression in articular cartilage

Authors: Cheng-Hai Zhang, Yao Gao, Unmesh Jadhav, Han-Hwa Hun, Kristina M Holton, Alan J. Grodzinsky, Ramesh A. Shivdasani, Andrew B. Lassar

List of Supplementary Materials:

Supplementary Figures 1-5

Supplementary Tables 1-10

Supplementary Data 1-2

Supplementary Figure 1

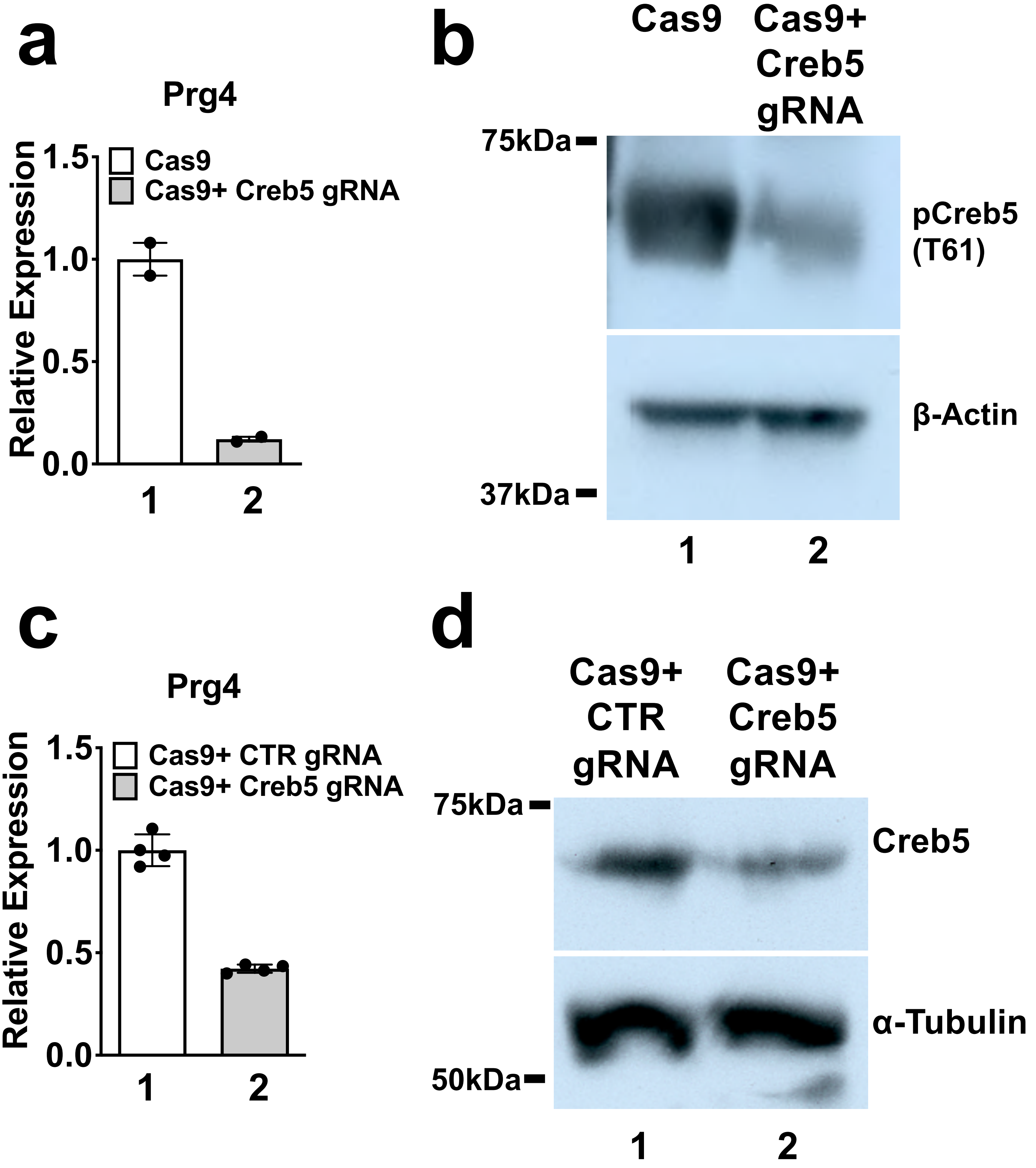

Supplementary Figure 1. CRISPR-Cas9 mediated mutation of the DNA binding domain of *Creb5* in SZCs decreases the levels of both Creb5 protein and TGF-β2-induced expression of *Prg4*. SZCs were infected with a lentivirus encoding either (a & b) Cas9 alone (lane 1) or Cas9 plus a *Creb5* guide RNA (targeting the DNA binding domain, lane 2) or (c & d) Cas9 plus a control guide RNA (lane 1) or Cas9 plus a *Creb5* guide RNA (lane 2). After selection in puromycin, to increase the efficiency of *Creb5* mutation, cells were cultured for at least an additional 14 days, prior to assaying *Prg4* expression and Creb5 protein levels. (a & c) Infected SZCs were cultured in ultra-low attachment dishes in the presence of TGF-β2 (20 ng/ml) for 3 days. Gene expression was assayed by RT-qPCR and normalized to *Gapdh*. Error bar indicates standard error of the mean (n=2 technical repeats for Supplementary Figure 1a, and n=4 technical repeats for Supplementary Figure 1c). Similar results have been obtained in 3 independent biological repeats. (b & d) Parallel SZC cultures to those displayed in (a & b) were cultured in the absence of TGF-β2 and total cell protein levels were assayed by immunoblot with antibodies directed against either phospho-Creb5 (T61), total Creb5, β-actin or α-tubulin, as indicated.

Supplementary Figure 2

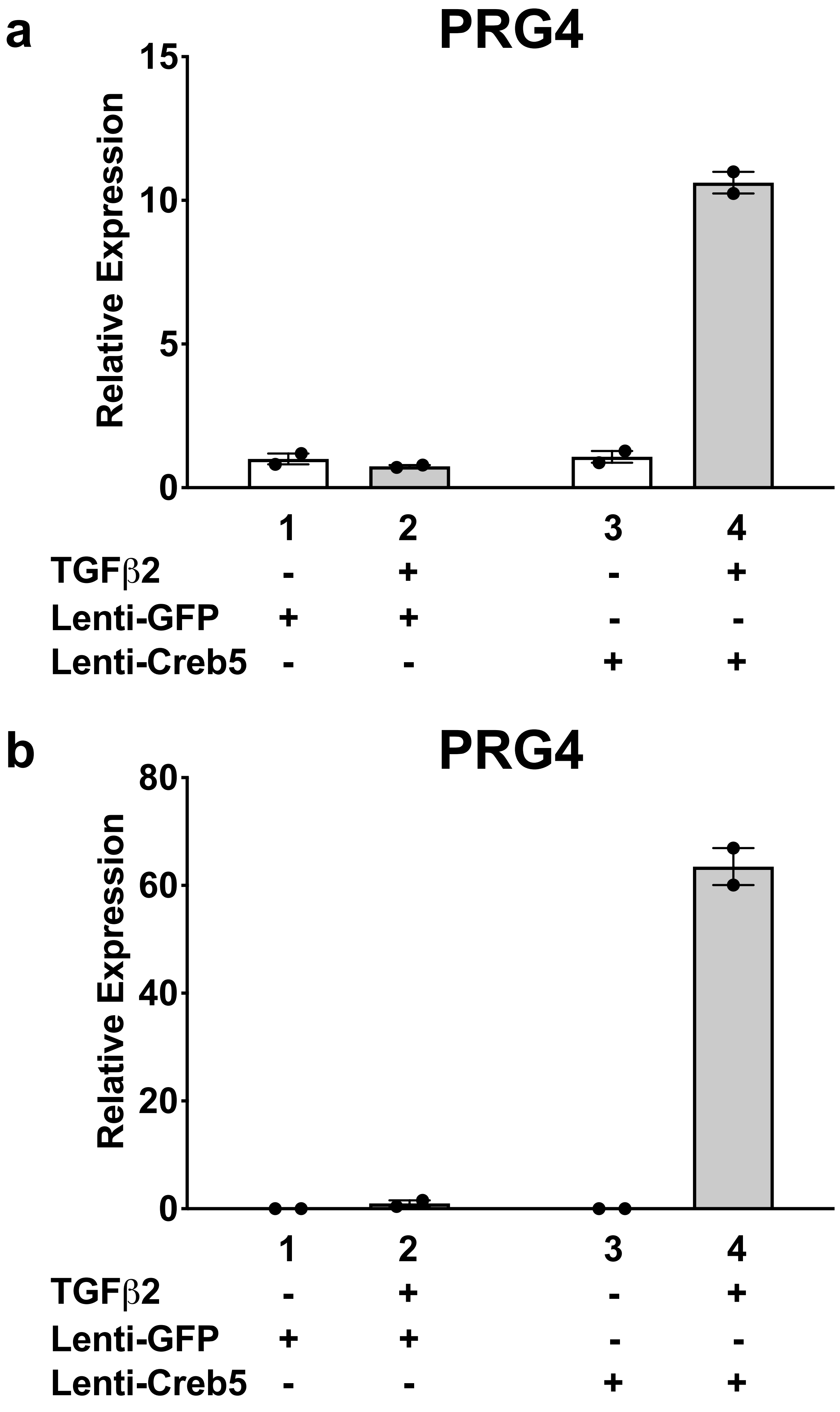

Supplementary Figure 2. Exogenous Creb5 can promote TGF-β dependent induction of *Prg4* expression in either a human chondrosarcoma cell line or in an immortalized human costal chondrocyte cell line. (a) Sw1353 human chondrosarcoma cells (ATCC HTB 94) or (b) immortalized human costal chondrocyte (C-28/I2; [49]) cells were infected with control lentivirus (Lenti-GFP) or Lenti-Creb5. After selection in puromycin, the cells were cultured in either the absence (white) or presence (grey) of TGF-β2 (20 ng/ml) for 3 days in ultra-low attachment plates. Gene expression was assayed by RT-qPCR and normalized to *GAPDH*. Error bar indicates standard error of the mean (n=2 technical repeats). Similar results have been obtained in 3 independent biological repeats.

Supplementary Figure 3

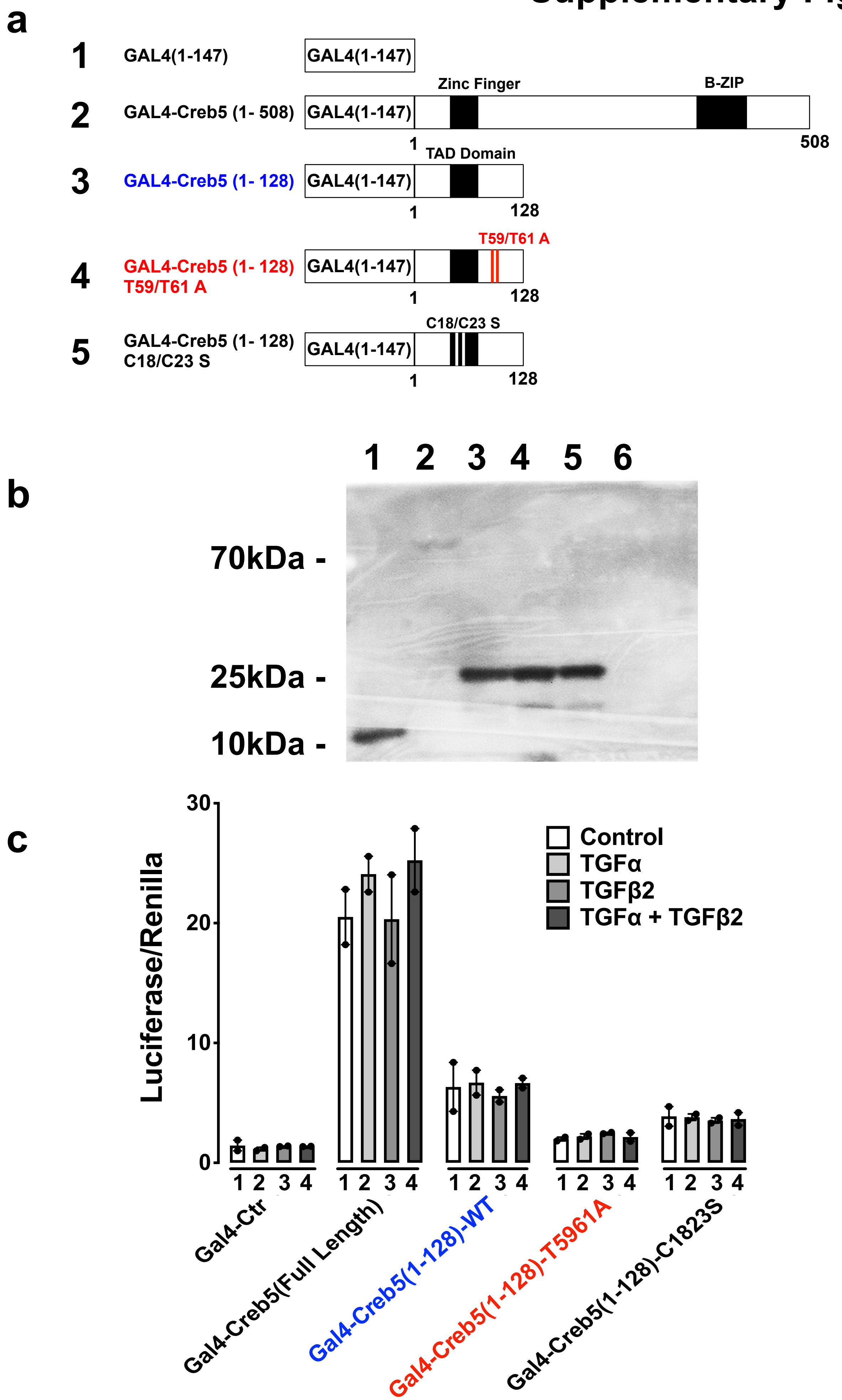

Supplementary Figure 3. The amino-terminus of Creb5 contains a relatively weak transcriptional activation domain, which requires both SAPK phosphorylation sites and an intact zinc finger domain for maximal activity. (a) Diagrams of GAL4 DNA binding domain fusions with either full length Creb5 (1-508) or with the N-terminus of Creb5 (1-128). Mutations in either the SAPK phosphorylation sites (T59/T61A) or in the zinc finger domain (C18/C23S) are displayed. (b) Expression of various GAL4 DNA binding domain fusions (as numbered and diagrammed in (a) in transfected 293T cells, as detected by a GAL4 western blot. Lane 6 is derived from non-transfected 293T cells. (c) Superficial zone bovine articular chondrocytes were transfected with various GAL4-Creb5 constructs, plus a GAL4-firefly luciferase reporter and a CMV-renilla luciferase reporter; cells were cultured in either the absence or presence of TGF-β2 (20 ng/ml) and TGF-α (100 ng/ml), as indicated for 3 days. Relative expression of firefly/renilla luciferase is displayed. Error bar indicates standard error of the mean (n=2 technical repeats).

Supplementary Figure 4

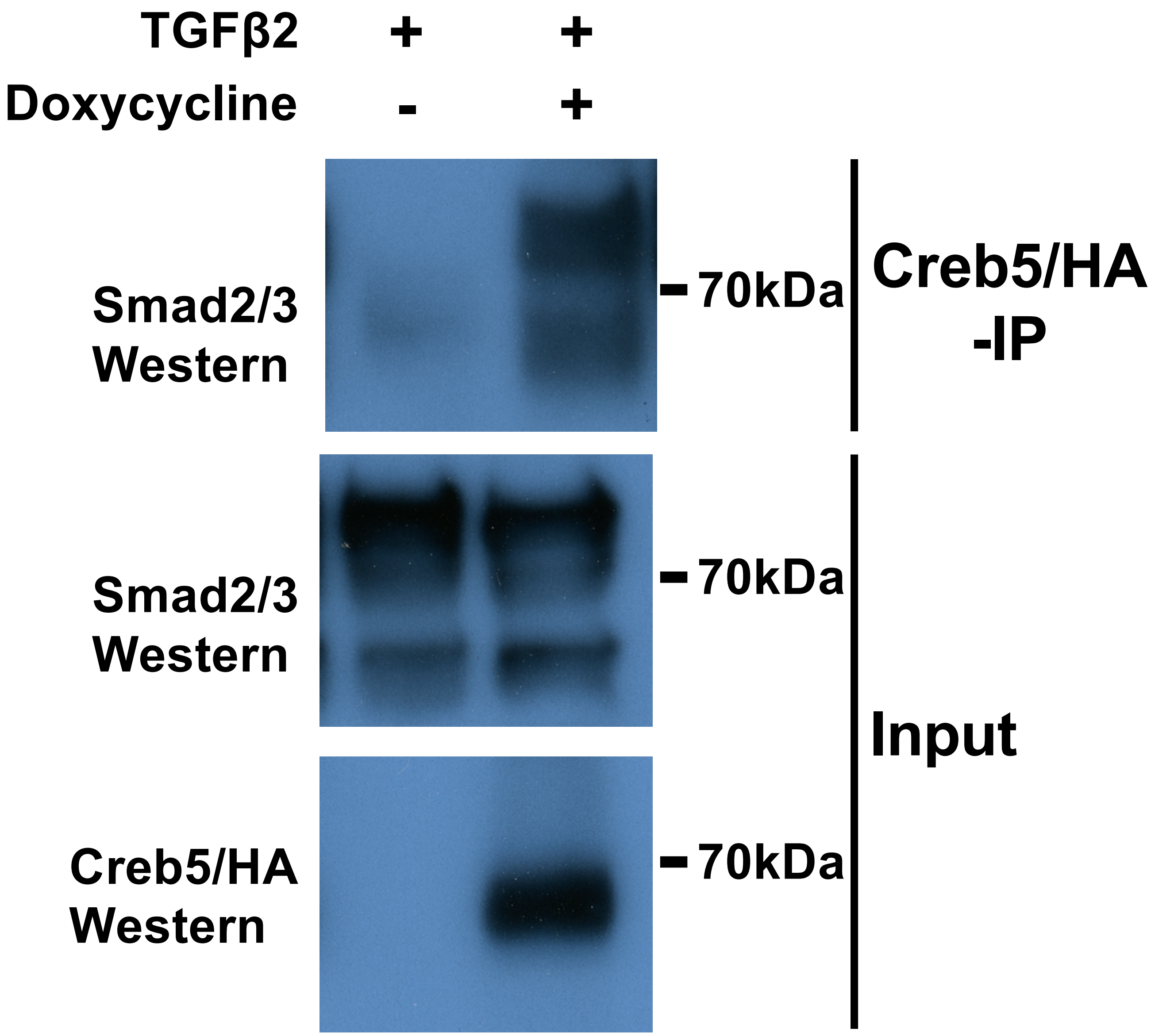

Supplementary Figure 4. Creb5 can co-immunoprecipitate Smad2/3. Deep zone bovine articular chondrocytes that were programmed to express doxycycline-inducible Creb5 lentivirus (DZC-iCreb5-HA) were cultured in ultra-low attachment dishes in the presence of TGF-β2 (20 ng/ml) with or without doxycycline (1μg/ml) for 3 days. After 3 days culture, cells were collected and co-immunoprecipitation was performed using monoclonal anti-HA agarose. Immunoprecipitated protein was detected using anti-Smad2/3 antibody in western blot. Input protein was detected using anti-Smad2/3 and anti-HA antibody.

Supplementary Figure 5

Uncropped blot images of the indicated figures (page 1of 8)

Figure 2a

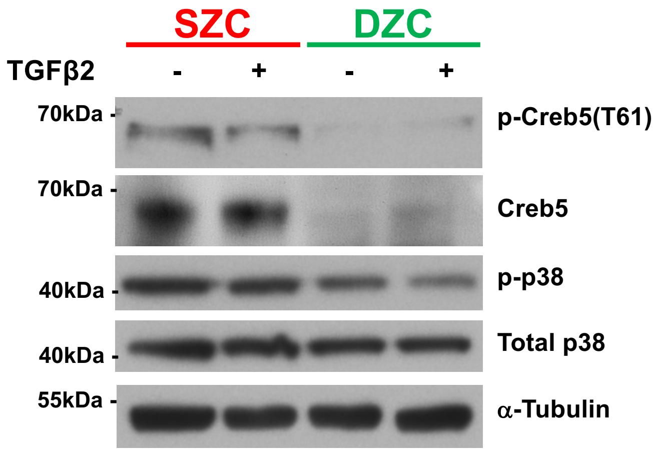

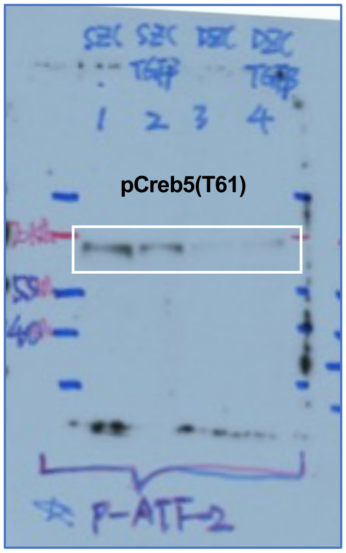

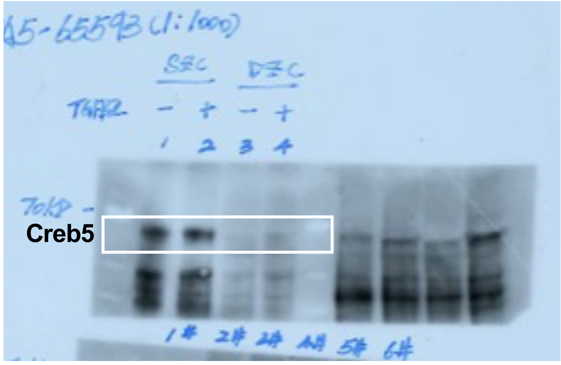

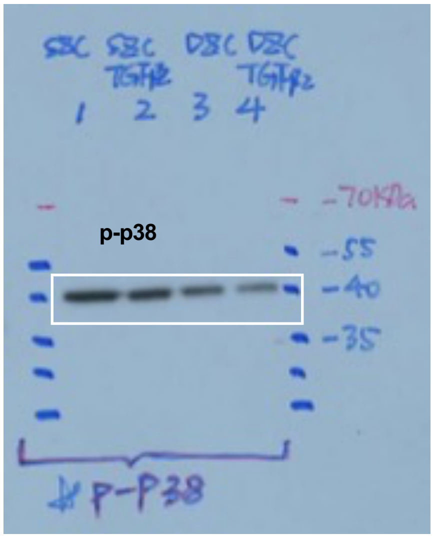

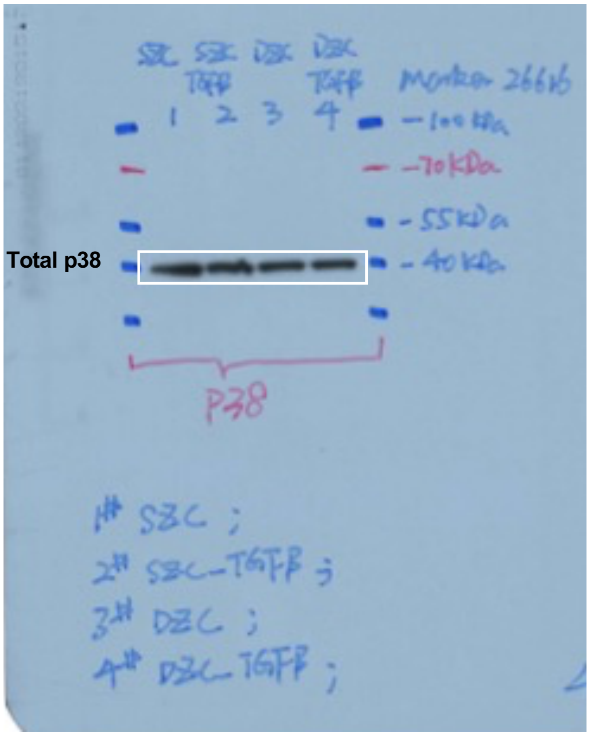

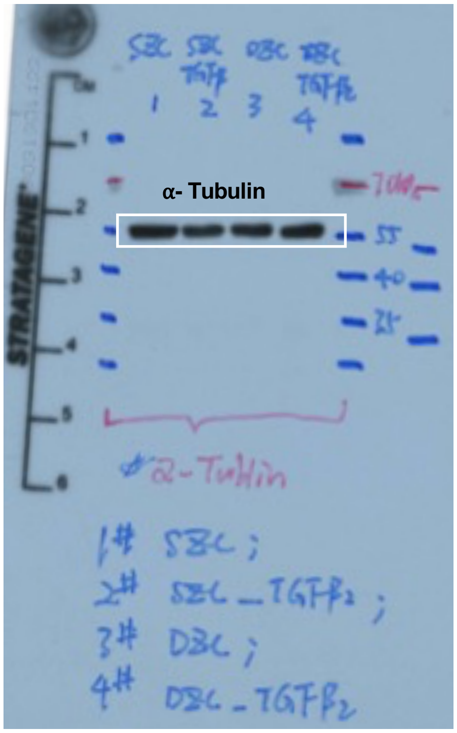

Uncropped blot images of the indicated figures (page 2 of 8)

Figure 3c

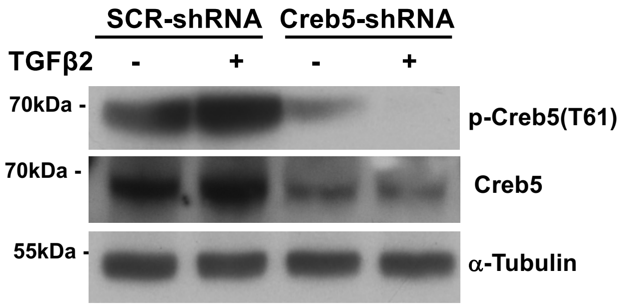

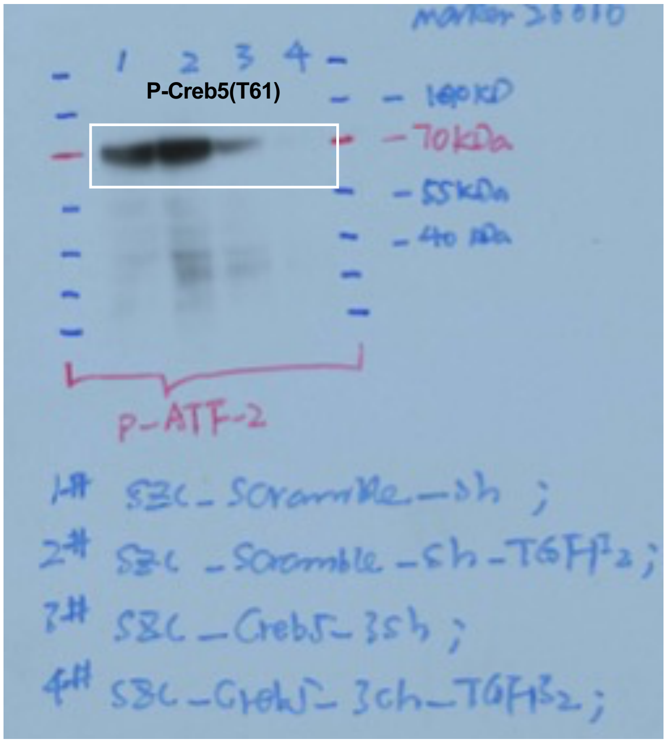

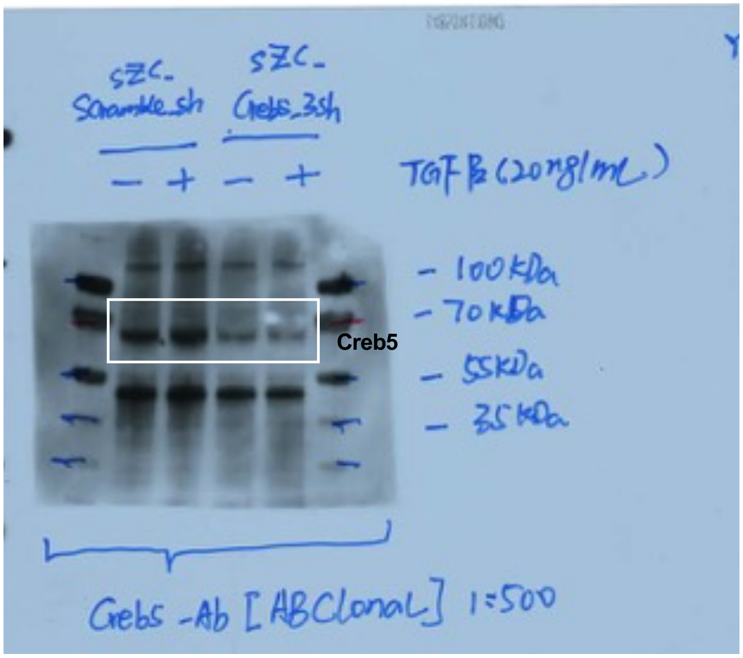

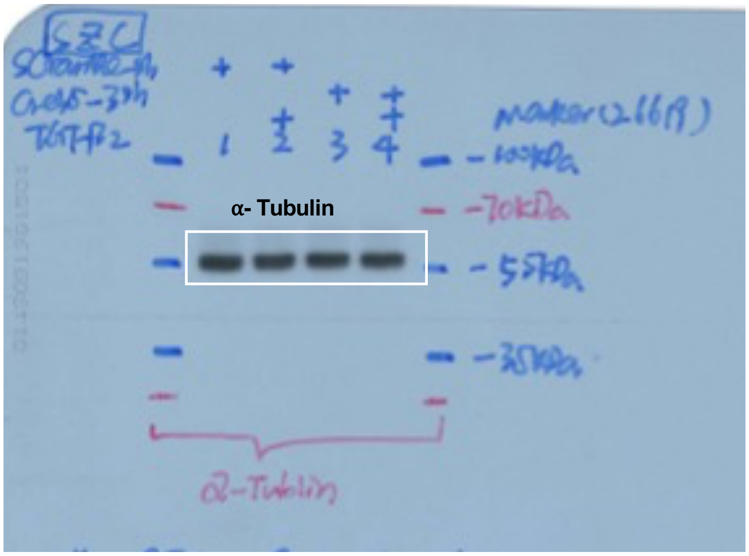

Supplementary Figure 5

Uncropped blot images of the indicated figures (page 3 of 8)

Figure 4b

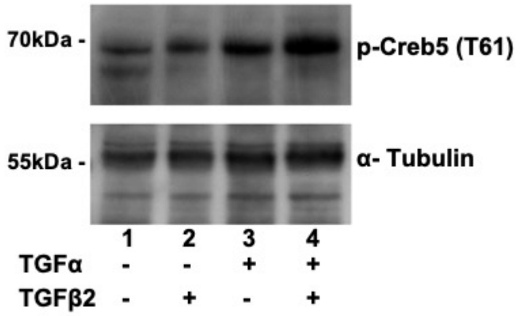

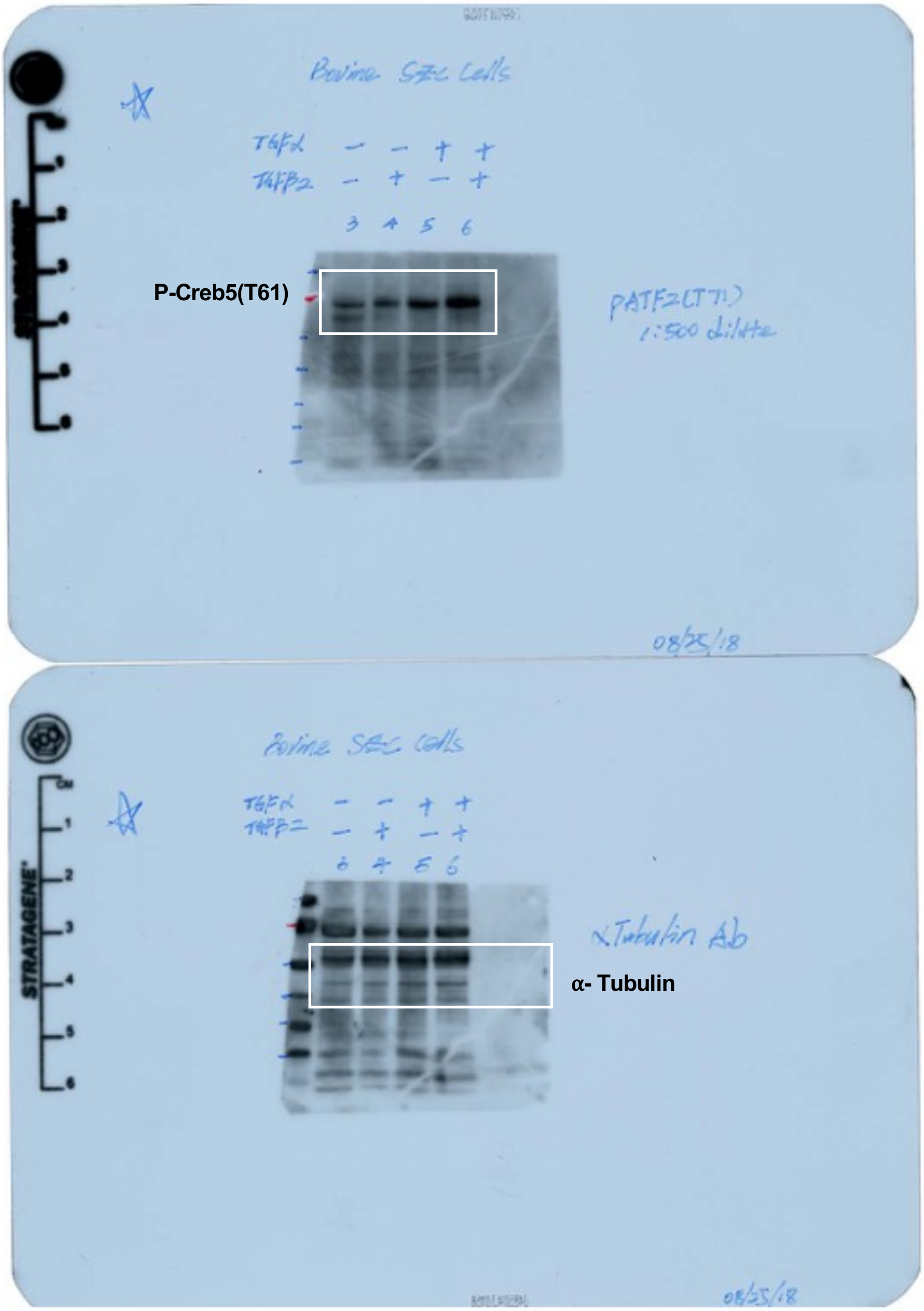

Supplementary Figure 5

Uncropped blot images of the indicated figures (page 4 of 8)

Figure 5c

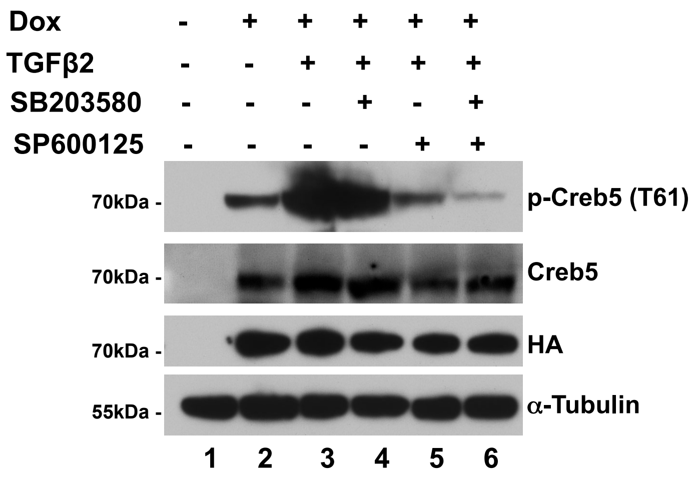

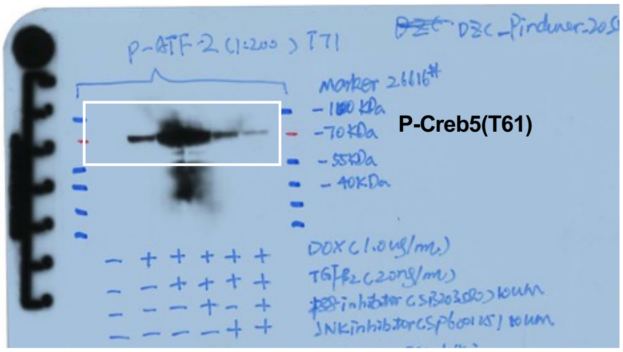

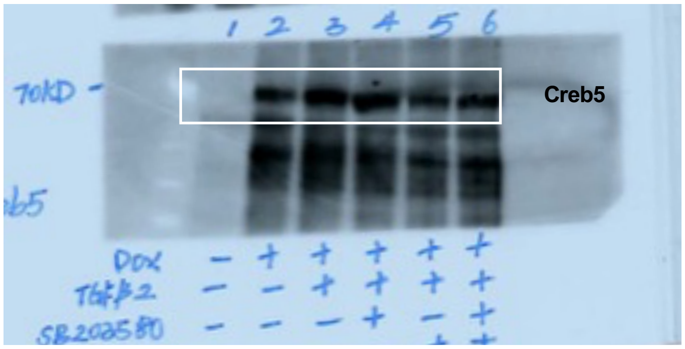

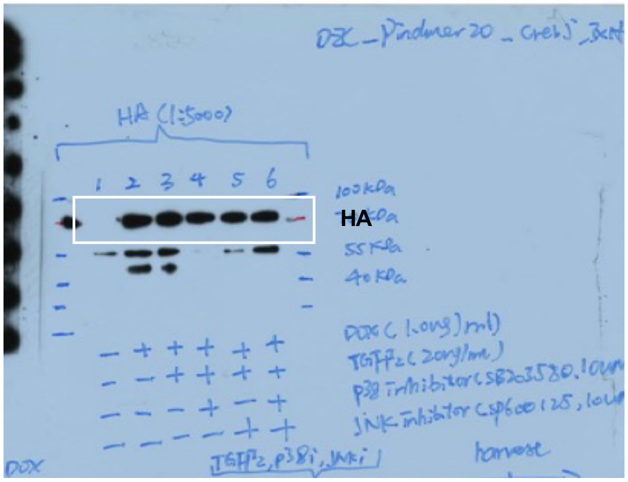

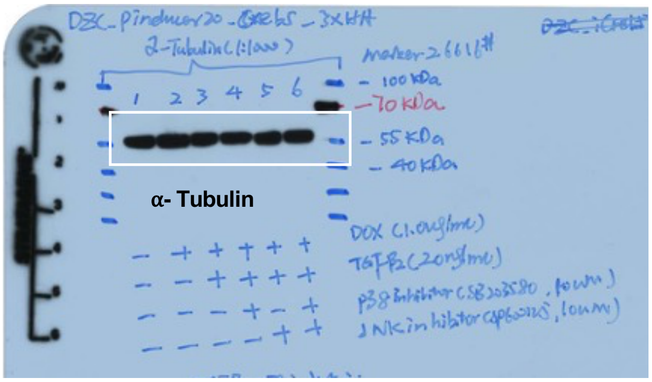

Supplementary Figure 5

Uncropped blot images of the indicated figures (page 5 of 8)

Figure 5d

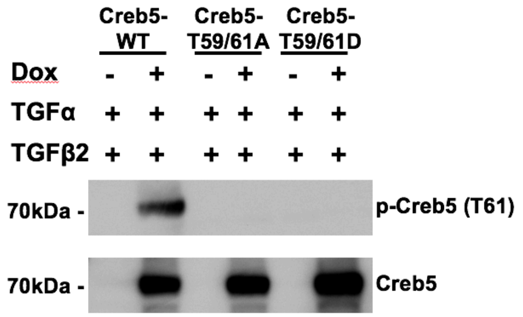

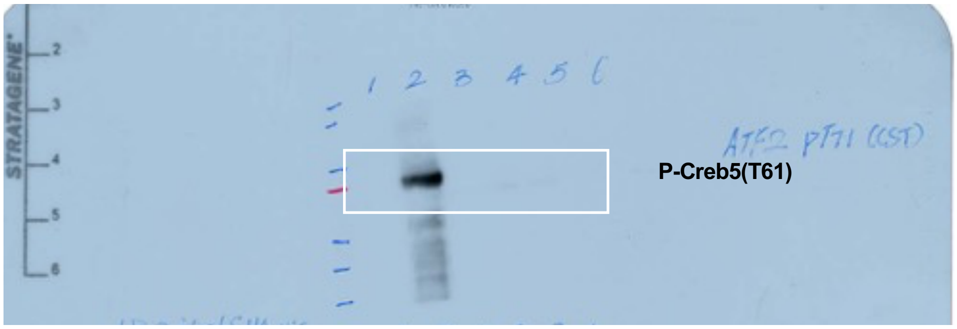

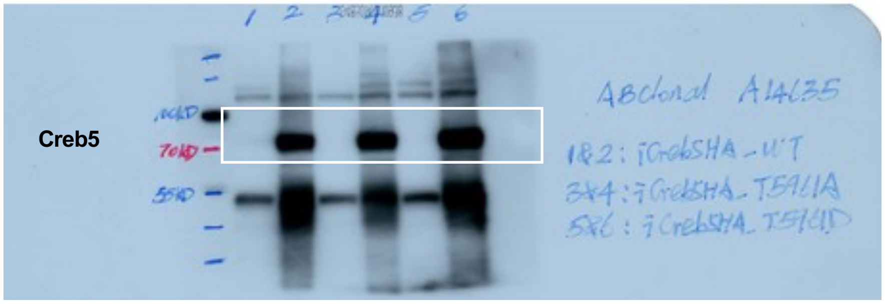

Supplementary Figure 5

Uncropped blot images of the indicated figures (page 6 of 8)

Supplementary Figure 1b

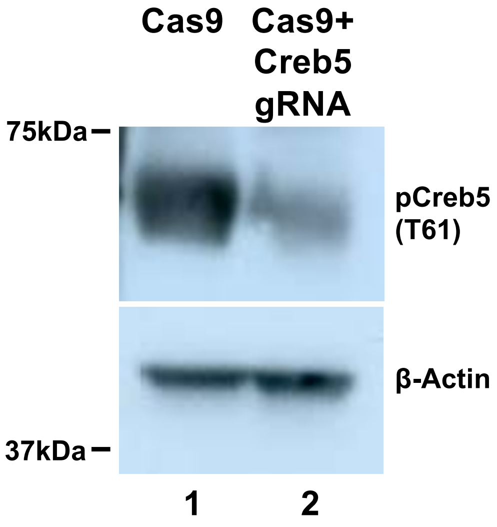

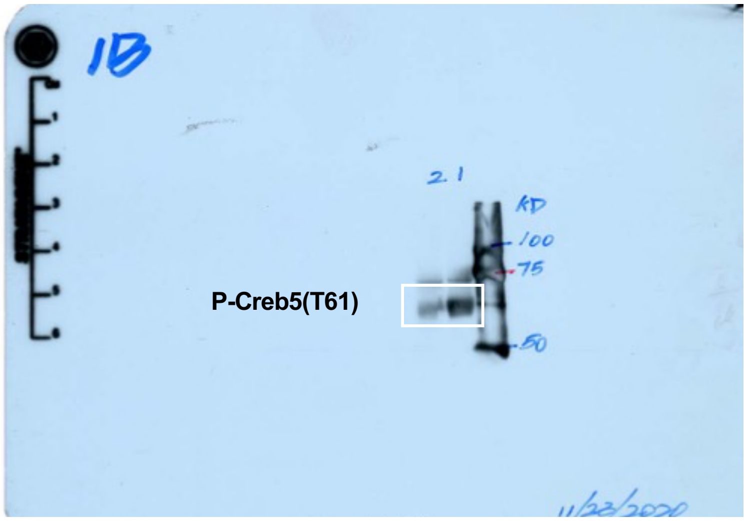

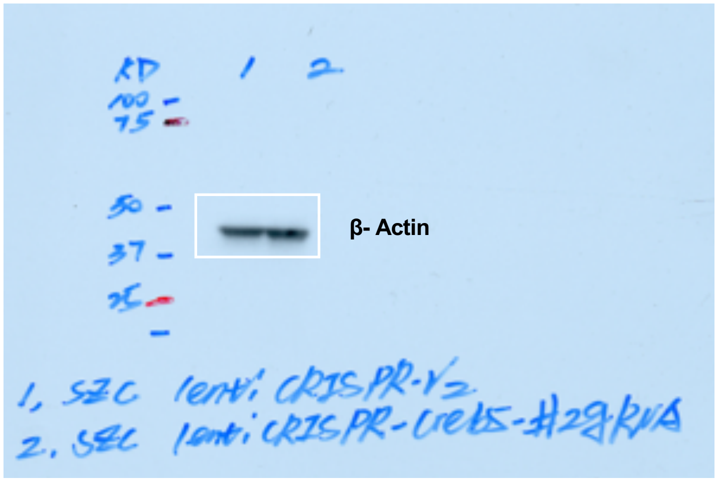

Supplementary Figure 5

Uncropped blot images of the indicated figures (page 7 of 8)

Supplementary Figure 1d

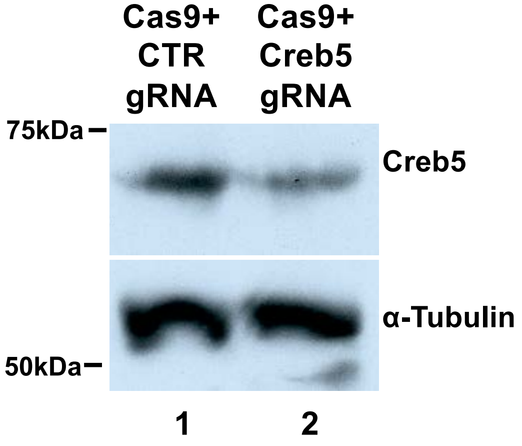

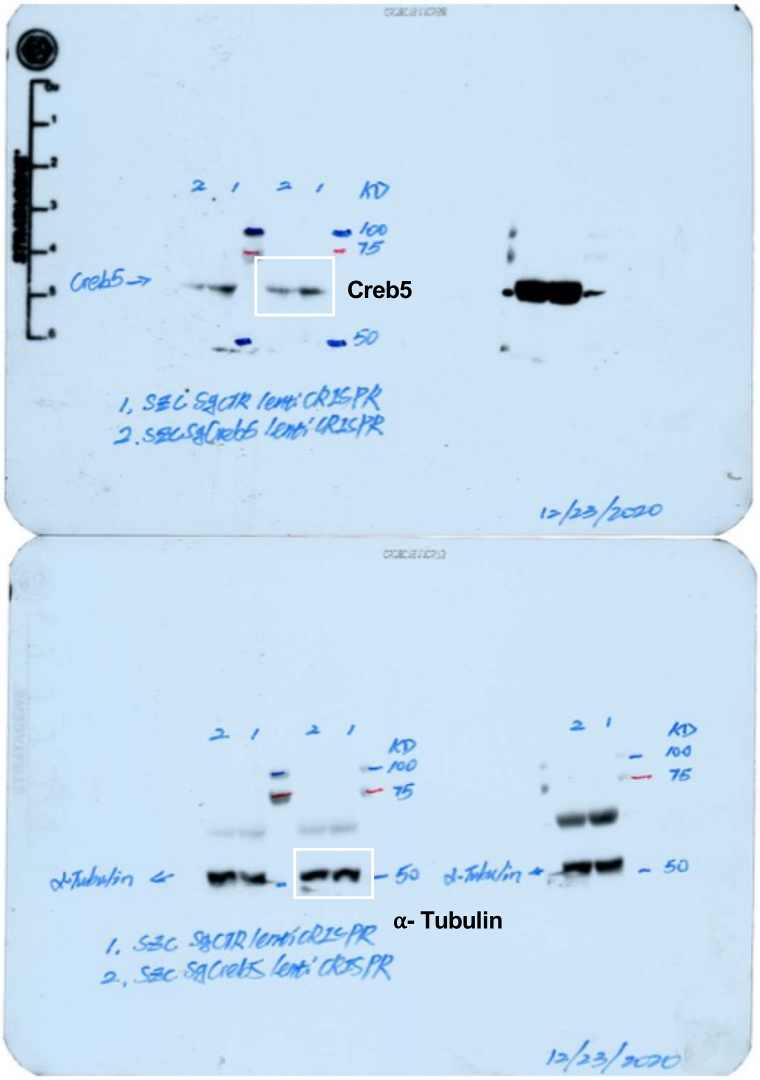

Supplementary Figure 5

Uncropped blot images of the indicated figures (page 8 of 8)

Supplementary Figure 4

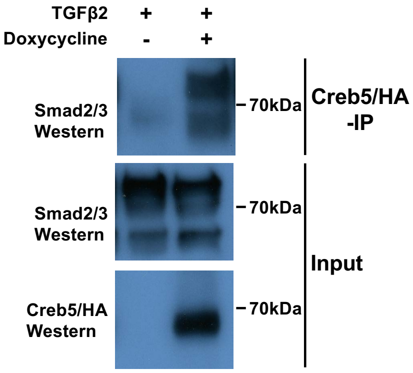

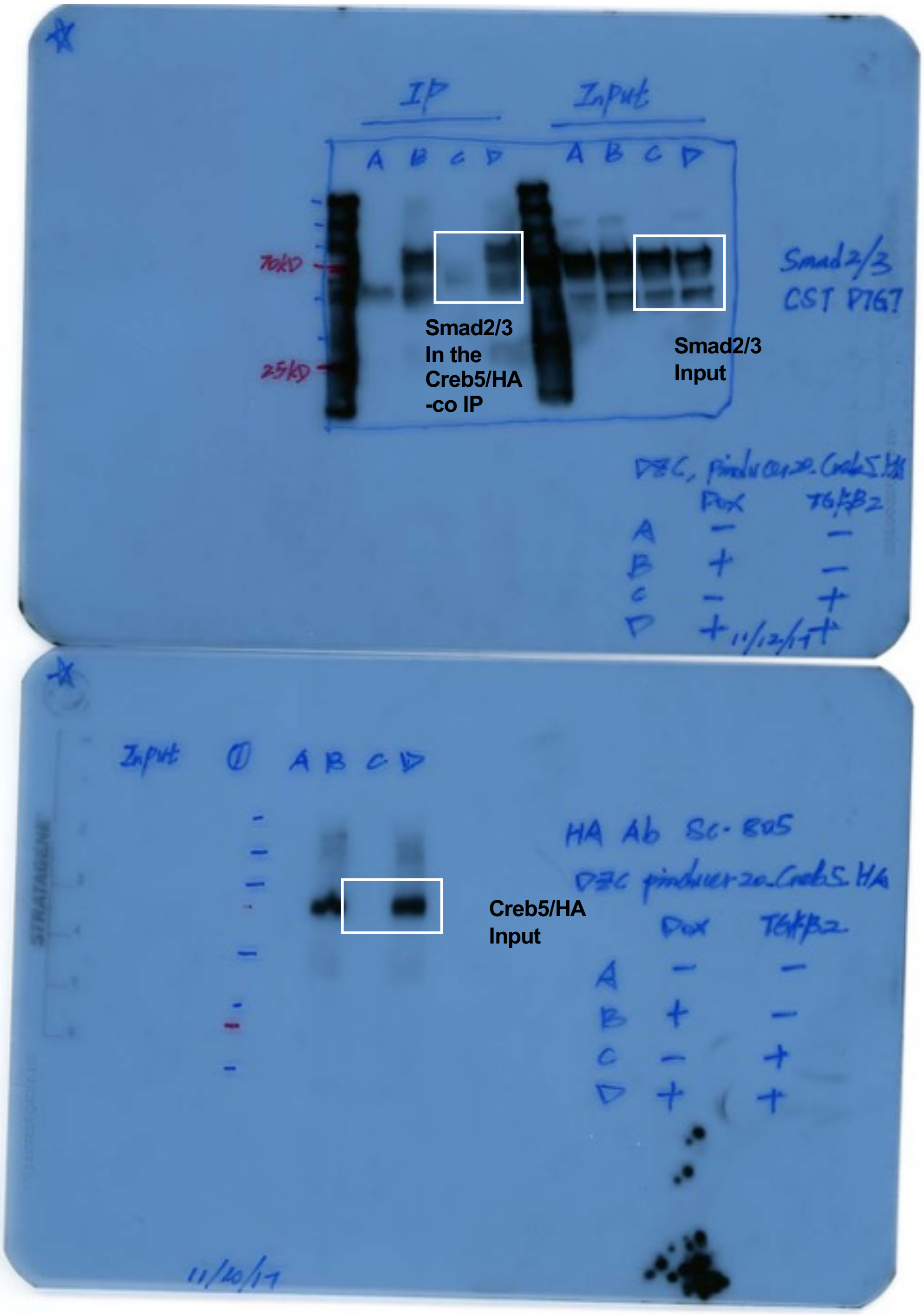

Supplementary Figure 5. Uncropped blot images of the indicated figures.

Supplementary Table 1 : RT-qPCR Primers to amplify bovine cDNA

Gene	Forward	Reverse
Prg4	CTTGATTCAGCAAGCTTCTTCTC	ACAGGAAAGCTCCACAGTGC
Creb5 (Primers 1) *Both primers are in the Creb5 coding region*	AGGCGGCGGAAATTTCTG	CATGGCTGTTATGGGGCA
Creb5 (Primers 2) *The reverse primer is in the Creb5 3' UTR*	AGACTGCCCCATAACAGCCATG	GCTCTTCTTGAAGGCAAGGTGC
EphA3	GCTGATGCTAGACTGCTG	CGGAAGGTAGTAATGTCG
Thbs4	ACACCAGTGACCAGGTCAGGCT	GGAGAAGCAGAACACGCCGA
Gapdh	TGGTGAAGGTCGGAGTGAAC	TGTAGACCATGTAGTGAAGGTCA

Supplementary Table 2 : ChIP-PCR Primers

	Forward	Reverse
***Prg4* Enhancer1**	AAAAACACTCTTTGCTGG	AGATGGTAAATTGCTTGG
***Prg4* Enhancer2**	AAGTGAGTTGTCGCTTAAA	AAACCAGTTGAACCATCT
***Prg4* Enhancer3**	AGGTACCACGGCTGTTAC	CAGTGTTTCAGACAGCAG
***Prg4* Enhancer4**	GTTTTCATTGGAAAGCATTC	CACAGCCAAAACAATTATC
***GAPDH* ATAC PEAK**	ACACAGGCGCTCCTGGGAAA	TTCCGCCCTCACGTCCAG

Supplementary Table 3: Prg4 Promoter and Enhancer E1,E2,E3,E4 sequences

**Promoter**	TAAAGGTGCTTGAAAAGATTTATAAGGATAGTAGTTGGAGAGTTTAGAAAAGA ATCACATTTATGCAATACACCTGCCGGAGGCTTATGAATTTATATTGAGAGTTA AGAGAATGTTGGAATGATAACTGTTAAATTAAATTTTACTAAAGCTGGTTACTT ATTTTTTCCCTTCAATATAGGATTTAAACCTTGTACTTTAAGAACATGCTATAAT TTGAATAGAGTTTTAAGATAAGTCTTTTTTTCTATTTGGGAGATTCTAAAGTATT AAAATGGTCAGTAATGTGGTTGAGATGGTGATTTGATGATTTTTTTTTTTCTGT ATAAGATTAAATCTAATGGTCAAGTATTTACAATGAATTAAGGCTGGTTATTTT TAAAAGTCTTGCTTTAAGAATGGGAAAGTTTTTTTTATCATTAAATTGAATTTTG AAATTATTGAAATGCTGTAATACAAGCATAAATTAGTGGTGAGATCCAAGAGGC GTTTCTAATACTTTTATTTTCTTTTCAGCGAGGGCTCAGTACCTGAAAACAGCC

**E1**	AGAAAACTTATGCCATAAGCTGAAACTAGATAAAGTTTCAAAAACACTCTTTGC<br>TGGGTTAACTGGAAAAAGAAAAAAAAAAAGAAATGATACCAGCATTTGACTATTT<br>TAAAGTTGTTGCACTAGTCAAAGCCTGCCAAGCAATTTACCATCTCACAGAACA<br>ATTTGTTCACTTCACTTAGAAATTAGAAGTGTGTTTGGGCCAATAAAGCTAAAT<br>TTGTAGTAGTCATTTTTATTAACGGAAGTGTTTTCCAGATGTTACTTAAGTCTG<br>GTTGTCTGGGACCTGTTCCAATAAATGAGGAAAGTTTGATTTCATAGGTTGTCA<br>CTGTTGATTCTGTCTAACCTTTGGACTAATTGGTTCATCCTAACATATTTGCTCC<br>GGTATATAAGAGCATTGGACATTGGAGAAATTCACCTCTCCTTTTACGGGTAAG<br>TGTCACAGTTAACTTGCTTCATGGAGTTATATGTTTTCAATTCAATAAGGATTTA<br>TTAAAAGTTTATT
**E2**	TTCAATTTTCCATTGGAGATACGTTTCTCTCTGTTTTCGCTCCAACCCAGGGCTC<br>ACTCCCTCTGCTTCCTTCTACCTTGCTTTACTATGCAGAATGCCTGAATGCTGGC<br>CAGCTTTTCCTTTGCTAAGGGCTTATGAAATTCTTATTTCAACCAAGCGGTCTCT<br>TTTGGGAAGGCCACATGGGAGTGCAAAGTGAGTTGTCGCTTAAAAAAATAAAT<br>TTTTCCGGATCATTGCATTACACAATCTGTAGTTTCTTGACTTTCCTGTGTCAAA<br>CTAATTTTTTAGATTCAAAAGCAGAGCCACAGATGGTTCAACTGGTTTCTCAAA<br>AGTTTTAAACAATCCCAACTTTAGTTTTACTGGCATTCGAATAAATTGCTCTGCC<br>TTTTCCCTCCTCTGGTATTCCTACCTGCCTTGTACTGAATAAAGATAGATGGTTC<br>TGTGTAAGACTGTGTCAACTATTTACGTCTAGATTTATCTTTTTTGGCTTTTTAC<br>TTCTTTTCTCCTTAAGTTCCCCACATTCCTTTTCTACAGGAAGCATGGAAAAGAT<br>AAAGGTGCCAGGAAAGATATTAGTTTTTGGCTGCTGGC
**E3**	AGGGTATGTCTTGCTTTCTCTTCCCAGGCATGCTTTTGAAAATCCTCCTGCCTCT<br>CTCTCCTTGACCAACAAGCCCTGTCTGCAGTCATGTTTCTCACTCACAGGAAGT<br>CAGTAATCCCTCAGGGGCTAGGGGTTTACTGTGTGTTCCTTCTTCAGAGAGTGG<br>GCAAACAAACGAATTCATCCTGCAGCAGCGATTGCCAGCTTTGAGCTGCCAGG<br>GTTTCCTGGAACAAGTCTGCTCAGTGTGTAATGCACCAGGCAACATCTAGAATC<br>CCTCCTCAGCAGGGAGCATTTTATAGGGTGATAGAGCCGACCCATCTTTGAAAT<br>TGTCTTTTCTGTTGGTGTTGCCTCAGGGTTTTCAGGGCTCTCTGCTTTTAGAGC<br>ACTGGCTCTAAGAGGTACCACGGCTGTTACAGTGATGAGTCATCATGACTCCAT<br>GGGAGTGTGGAGGAGACCAAGGATGAGTCAGTAGTCTGTCTGTTCATTCACAT<br>CTCATCTTGTTTACATATTCTGCTGTCTGAAACACTGTCTTTTCTGGTTTTGAAT<br>AAACTGCTTTGTTCTCTCCCTCCCCTTACAGAAACATTGCACTCCGTTTAGGTAC<br>CAAGTTGGGGGGGTGCTTGGAAAATGAGATAATGGCCATCGTGAATAGGCTCC<br>ACTAATAAGGAGGTAGTTCTGAAACCGAACTCTGGTTGGATCTCACTTTGTTTT<br>CCTCTCAGTGGTGACTCCCATGTTACTGTAACATGGTCAAGTGTGCGCTGCCCA<br>GGATTCATCCAAAACCTATTTGTAATCTGTCTGAGGGGAATAGGGA
**E4**	GGAAAACTATTAATCAGCATAGTGGAGGAACATTTCTTAACTCTTCAATGTTTT<br>CATTGGAAAGCATTCCAAAAATAATGGAGACAACACCCTTGTAATTAAAATCTC<br>CAAGTGCAGAGTTCCTGCAGCTTTCAGATGGACCTATGAGATGACCTTCTTATT<br>CATTCATCACTCTGATAATTGTTTTGGCTGTGAGACAAAAAACTAAGAATTTAT<br>TTTGGGCAGAGGATGGATCGGGAGGGAAGATGATTAGAGAATGATCAATCATT<br>GAGAGAACCATATTAATAGAGGCAATAGTTTATGCCACCAGGGGCAGGTCAGA<br>TTACTTGAG

Supplementary Table 4: Sequence of dCas9-KRAB guide RNAs that target the bovine Prg4 enhancers

**E1-1#gRNA**	G CTC CGG TAT ATA AGA GCA T
**E1-2#gRNA**	T CAT CCT AAC ATA TTT GCT C
**E2-1#gRNA**	G ATT GTG TAA TGC AAT GAT C
**E2-2#gRNA**	A AGG CAG GTA GGA ATA CCA G
**E3-1#gRNA**	TTAGTGGAGCCTATTCACGA
**E3-2#gRNA**	AGACAATTTCAAAGATGGGT
**E3-3#gRNA**	ACTCCGTTTAGGTACCAAGT
**E4-1#gRNA**	A TTT TGG GCA GAG GAT GGA T
**E4-2#gRNA**	GTAATCTGACCTGCCCCTGG

Supplementary Table 5: Antibodies employed for Western Blots

Cat. No.	Concentration	Target	Host	Vendor
PA5-65593	1:1000	Creb5	Rabbit	Thermo Fisher
9221S	1:500	p-Creb5	Rabbit	Cell Signaling Technology
9211S	1:1000	p-p38	Rabbit	Cell Signaling Technology
9212S	1:1000	P38	Rabbit	Cell Signaling Technology
T9026	1:1000	α-Tubulin	Mouse	Sigma
ab9110	1:1000	HA	Rabbit	abcam
sc-805	1:1000	HA	Rabbit	Santa cruz biotechnology
A14635	1:1000	Creb5	Rabbit	ABclonal
sc-510	1:500	GAL4	Mouse	Santa cruz biotechnology
8685S	1:1000	Smad2/3	Rabbit	Cell Signaling Technology
A5316	1:5000	β-Actin	Mouse	Sigma

Supplementary Table 6: Antibodies employed for either ChIP or co-immunoprecipitation (Co-IP)

Cat. No.	Concentration	Target	Host	Vendor
ab9110	5 µg per ChIP	HA	Rabbit	abcam
ab46540	5 µg per ChIP	IgG	Rabbit	abcam
A2095	25 µl per Co-IP	HA	Mouse	Sigma

Supplementary Table 7: Antibodies employed for immunohistochemistry

Cat. No.	Concentration	Target	Host	Vendor
PA5-65593	1:100	Creb5	Rabbit	Thermo Fisher
MABT401	1:50	Prg4	Mouse	EMD Millipore
11867423001	1:100	HA	Rat	Sigma
AB5535	1:100	Sox9	Rabbit	EMD Millipore
18338S	1:50	Phospho-Smad2 (Ser465/Ser467)	Rabbit	Cell Signaling Technology
A11029	1:250	anti-Mouse IgG; Alexa Fluor 488	Goat	Thermo Fisher
A11037	1:250	anti-Rabbit IgG ; Alexa Fluor 594	Goat	Thermo Fisher
A11006	1:250	anti-Rat IgG; Alexa Fluor 488	Goat	Thermo Fisher
S11227	1:250	Streptavidin, Alexa Fluor™ 594 conjugate		Thermo Fisher

Supplementary Table 8: Growth factors and inhibitors used for chondrocyte culture

	Cat. No.	Concentration	Vendor
TGF-α	239-A-100	100 ng/ml	R&D Systems
TGF-β2	302-B2-010	20 ng/ml	R&D Systems
Doxycycline	D9891-1G	1 µg/ml	Sigma
SB203580	152121-47-6	10 µM	TOCRIS
SP600125	129-56-6	10 µM	TOCRIS

Supplementary Table 9: Target sequence (sense strand) of in situ hybridization probes.

**Prg4**	AGATTCAGAACAAACCTGAAGAAACAACTCCTGCATCAGAAGATTCTGAT<br>GATTCTAAAACAACTCTAAAACCACAGAAGCCAACCAAAGCACCCAAGCC<br>TACCAAAAAGCCAACCAAAGCACCCAAGAAGCCCACCTCTACCAAAAAGC<br>CAAAGACACCAAAAACAAGAAAACCAAAAACTACACCAGCTCCTCTAAAG<br>ACGACTTCAGCAACACCTGAACTGAATACCACCCCTCTAGAAGTCATGCT<br>GCCAACCACCACCATCCCTAAACAAACTCCAAACCCTGAAACAGCTGAAG<br>TAAATCCAGATCATGAAGATGCAGATGGAGGTGAAGGAGAAAAACCTCT<br>GATTCCCGGGCCCCCTGTGCTATTCCCCACAGCTATTCCAGGCACTGATC<br>TTTTGGCCGGGAGACTCAATCGAGGCATTAACATCAATCCCATGCCTTCA<br>GATGAGACCAATTTATGCAATGGTAAGCCAGTGGATGGACTGACTACGCT<br>GCGCAATGGGACATTAGTTGCATTTCGAGGTCATTATTTCTGGATGCTGA<br>ATC
**Murine Creb5**	GCCTGTCCCAGGCTCTCTATCATCTCTACTCCATCTCCACAACAGACAGAG<br>GCAGCCCATGCCGGCCTCCATGCCTGGAACCCTGCCCAACCCCACCATGC<br>CAGGATCTTCTGCCGTCTTGATGCCTATGGAGAGACAGATGTCAGTGAAC<br>TCCAGCATCATGGGCATGCAAGGTCCAAACCTCAGCAACCCCTGTGCTTC<br>TCCCCAAGTCCAGCCAATGCATTCAGAAGCCAAAATGAGACTGAAGGCTG<br>CGCTGACTCACCATCCTGCCGCCATGTCGAACGGGAACATGAGCACCATC<br>GGACACATGATGGAGATGATGGGCTCCCGGCAAGACCAGACACCGCACC<br>ACCACCTGCACTCACACCCGCATCAGCACCAGACACTGCCGCCCCACCAC<br>CCCTACCCACACCAGCACCAGCACCCCGCACACCATCCCCACCCACAGCCT<br>CACCACCAGCAGAACCACCCGCACCACCACTCCCATTCCCACCTTCACGCA<br>CACCCGGCGCACCACCAGACCTCGCCACACCCACCCCTGCACACCGGCAA<br>CCAAGCACAGGTTTCACCAGCTACACAACAGATGCAGCCAACCCAGACAA<br>TACAACCACCCCAGCCCACAGGGGGACGCCGGCGAAGAGTGGTGGATGA<br>GGACCCAGATGAGAGGCGGAGGAAATTTCTGGAAAGGAACCGGGCCGCC<br>GCCACCCGCTGCAGACAGAAGAGGAAGGTCTGGGTGATGTCACTGGAAA<br>AGAAAGCTGAAGAGCTCACCCAGACAAACATGCAGCTTCAGAATGAAGT<br>GTCCATGTTGAAAAACGAGGTGGCCCAGCTGAAGCAGTTGTTGTTAACAC<br>ATAAAGACTGTCCGATAACAGCCATGCAGAAAGAATCCCAAGGGTATTTA<br>AGTCCAGAGAGCAGCCCTCCTGCGAGCCCTGTGCCAGCATGCTCTCAGCA<br>GCAAGTTATCCAACACAACACCATCACTACATCCTCATCGGTCAGCGAGG<br>TCGTGGGGAGCTCCACCCTCAGTCAGCTTACAACTCACAGAACAGACCTG<br>AATCCTATTCTTTAAAAGGCATCGGTCAAACCTGGCCTTTGAGAAGAGCT<br>GTAGCATGCCGTACATCCTTTCTCAAAGGGGCATTTTTTTAGAATTATCT<br>CAGACCTGGAAGACGCCTCAGCCCTTCAAAGACTGGCTTTCATTTTTATA<br>GTTATTATGGAAATGTTGTCTTTTATACTTAGTTATATAAGAAAAAAGGG<br>AGATATGCAATGAATATCTATCAGCTTGGGGAGCACGTTGGTGCTTCTCT<br>GCAATTTTCTGGTACCAGTTTCTTGTTTATAAACGGAACCTTTCCTGTATA<br>TAGCCATGGTTTCATTCTTACCAGCCCAACCCTTTGCCTGGAACAATGAAT<br>CTTGTTCAACTACAGCTTTTAGCCAAAATGAGGTATGCTTAGATGTCAAG<br>CGAGATGGATCCACACAGTAACTGGGTGGGAAAGCTCATGATGTCATAA<br>CTCATGTTGAGTTTGTGCTGTGATGTCACCAGAATCTCAGATAAACACAT<br>GGGCCTTCCTGAATATTTTTTCTCTTGCTAGAAAAAAATAAATTATGGTCC<br>ATCCATATCCCATGAAAGCCACCAAGCATCTCAGGCCCCCTCCTTCCTCTC<br>TTTTCTACTTGTGCAGATGTCCAATATCCATCTCATTTTCTTTTCCCCGGA<br>TCCCTTGTTTACTCTTTGTTTTGACTTTTTTTCTGTTTCCTTTTCTCCCCTT<br>TAGCTTTGCATGTAAAAAAGAAAATAATGTTTAAAGAAGAGAAAAAGCAA

	ATCTGGAAACTGTGGACCTAGCCACAGTTTAACCCACAGCTGGAGTTCAT<br>TCAATTTTTGCCTTTCACAAAATAGCAACCAGGAGATGTTTAATGTGCCT<br>GATTTAATGTTTTTAATAACCAGAGCAAATAAAAGGTGGTTTGGTTATAG<br>GTGAAGCACTGTTGAATGCCAGCTGTGGGGACACTAGGGAAAGGGACTT<br>CGTAAGCTCCAACTGTGAAAATTCAAATAAGGATGTGGGCTCTAACATCA<br>CACCCTCGAATTACAGCTCGCTTCTATGGCCTGTCTATAATGTAAAAAATC<br>CATGCACTATATAATAGTTCAGAAGGGCTCTGTTCACTACACAGATTACA<br>TTGTTCAATCATCAGCTGCTAATAACCTAAGATTTATTATTATTATTTTTC<br>TTAAGCCTATGGAACCAGCTCTGCTGTTCTGGTGGGCAAAAGCAAACTCA<br>CTCTTGGAGCAACAGAGAGAAAGCGAGGCCAGCGTTTCTCGGGGACTCG<br>CAGTCTGCCAGAACAGTCAGACTCCTTGGCTGCTGACCGAGTCCCATGGA<br>GGTGGCCAGGCTGGTGCGCTCATCTGAGTAGTTCTGATTTATATTTTCAG<br>CAATGTCCACGGACTTGCCCATTACAGAAAGCAGATCAAACCCAAACCAC<br>AGTTGTGCCTCCTTGAAACAAGCCATTCTACTCTGCTGGTGTTTTACTATC<br>GTGTTTCACAAATAATAGGGGCTAATGTTTCTCACTAGCAGTCTGGGCAT<br>ATGCTGGTGTTTCATCTCTGCCCAAATAATTCACCTCCTAACCTATGTGTG<br>TGTGTGTGTGCACATGGATGTGTGTGCCTGAGTGTGTGAGTGTGTGTGT<br>GTGTGTGTGTGTGTGTGTGTGTGTTTATGAACAGTATAGGTTTTAAAAGA<br>ACAGTATTTTACAAAAGCCATCACTTTTATAAGAGTTCTGTAAAGGAAGG<br>ATGTACTTCTTCGCTCACTATAGTTTAAAAAAATTCTATTTTAGAGGAAAA<br>AAAAAAAAAAAAAAAA
**Col2a1**	CCTGTCTGCTTCTTGTAAAACCCCCGAACCCTGAAACAACACAATCCATTG<br>CGAACCCAAAGGACCCAAACACTTTCCAACCGCAGTCACTCCAGGATCTG<br>CACTGAATGGCTGACCTGACCTGATGATACCCAACCGTCCTCCCCTCACA<br>GCCCGGACTGTGCTCCCCTTTCTAAGAGACCTGAACTGGGCAGACTGCAA<br>AATAAAATCTCGGTGTTCTATTTATTTATTGTCTTCCTGTAAGACCTCTGG<br>GTCCAGGCGGAGACAGGAACTATCTGGTGTGAGTCAGACGCCCCCCGAG<br>TGACTGTTCCCAGCCCAGCCAGAAGACCCCTACAGATGCTGGGCGCAGG<br>GACTGCGTGTCCTACACAATGGTGCTATTCTGTGTCAAACACCTCTGTAT<br>TTTTTA
**Matrilin1**	GGATCCAAGAGCGTGCGGCCTGAGAACTTTGAGCTGGTGAAGAAGTTCA<br>TCAACCAGATTGTGGACACGTTAGATGTGTCGGACAGGCTAGCCCAGGT<br>GGGGCTGGTGCAGTACTCCAGCTCCATTCGCCAGGAGTTCCCACTCGGCC<br>GCTTCCACACCAAGAAGGACATTAAGGCCGCGGTGCGGAACATGTCCTAC<br>ATGGAGAAAGGCACCATGACTGGCGCCGCCTTGAAGTATCTCATAGATAA<br>TTCTTTCACTGTGTCCAGCGGGGCAAGGCCTGGAGCCCAGAAGGTGGGC<br>ATCGTCTTCACCGATGGCCGGAGCCAGGACTACATTAATGACGCTGCCAG<br>GAAGGCCAAGGACCTTGGCTTTAAGATGTTTGCGGTGGGCGTGGGCAAT<br>GCTGTGGAGGAAGAGCTGAGGGAGATCGCTTCCGAGCCCGTGGCAGACC<br>ACTACTTTTACACAGCTGACTTCAAGACCATCAACCAGATTGGCAAGAAG<br>CTGCAGAAACAAATCTGTGTGGAGGAAGACCCCTGTGCTTGTGAGTCCAT<br>ACTGAAATTTGAGGCCAAGGTGGAGGGTCTGCTGCAGGCCCTGACCAGG<br>AAGCTGGAAGCTGTGAGCGGGCGGCTGGCTGTCCTGGAGAACAGAATCA<br>TCTAA

Supplementary Table 10: PCR primers for human *PRG4* and *GAPDH*

Gene	Forward	Reverse
PRG4	TGTGACTGCGACGCCCAATGTA	GGTTTGAGATGCTCCTGAAGGTG
GAPDH	GTCTCCTCTGACTTCAACAGCG	ACCACCCTGTTGCTGTAGCCAA
