# Supplementary material for: Creb5 establishes the competence for Prg4 expression in articular cartilage
Source: Commun Biol. 2021 Mar 12;4:332. doi: 10.1038/s42003-021-01857-0 (PMC7955038; doi:10.1038/s42003-021-01857-0)
Supplement: Supplementary file 2 — Description of Supplementary Files [file 42003_2021_1857_MOESM2_ESM.pdf]

Description of Additional Supplementary Files

File Name: Supplementary Data 1

Description: **List of genes that are differentially expressed in superficial versus deep zone bovine articular chondrocytes.** 320 genes are listed whose expression differed (by indicated LogFC) in superficial versus deep zone articular chondrocytes (false discovery rate, FDR <0.05).

File name: Supplementary Data 2

Description: Primary data for graphs and charts
