# Supplementary material for: Creb5 establishes the competence for Prg4 expression in articular cartilage
Source: Commun Biol. 2021 Mar 12;4:332. doi: 10.1038/s42003-021-01857-0 (PMC7955038; doi:10.1038/s42003-021-01857-0)
Supplement: Supplementary file 5 — Reporting Summary [file 42003_2021_1857_MOESM5_ESM.pdf]

nature research

Corresponding author(s): Andrew B. Lassar

Last updated by author(s): Jan 19, 2021

Reporting Summary

Nature Research wishes to improve the reproducibility of the work that we publish. This form provides structure for consistency and transparency in reporting. For further information on Nature Research policies, see Authors & Referees and the Editorial Policy Checklist .

Statistics

For all statistical analyses, confirm that the following items are present in the figure legend, table legend, main text, or Methods section.

n/a	Confirmed	
☐	☒	The exact sample size (n) for each experimental group/condition, given as a discrete number and unit of measurement
☐	☒	A statement on whether measurements were taken from distinct samples or whether the same sample was measured repeatedly
☐	☒	The statistical test(s) used AND whether they are one- or two-sided<br>*Only common tests should be described solely by name; describe more complex techniques in the Methods section.*
☒	☐	A description of all covariates tested
☐	☒	A description of any assumptions or corrections, such as tests of normality and adjustment for multiple comparisons
☐	☒	A full description of the statistical parameters including central tendency (e.g. means) or other basic estimates (e.g. regression coefficient) AND variation (e.g. standard deviation) or associated estimates of uncertainty (e.g. confidence intervals)
☐	☒	For null hypothesis testing, the test statistic (e.g. F, t, r) with confidence intervals, effect sizes, degrees of freedom and P value noted<br>*Give P values as exact values whenever suitable.*
☒	☐	For Bayesian analysis, information on the choice of priors and Markov chain Monte Carlo settings
☒	☐	For hierarchical and complex designs, identification of the appropriate level for tests and full reporting of outcomes
☒	☐	Estimates of effect sizes (e.g. Cohen's d, Pearson's r), indicating how they were calculated

Our web collection on statistics for biologists contains articles on many of the points above.

Software and code

Policy information about availability of computer code

Data collection	Raw RNA-seq reads were assessed with FastQC 0.11.3 followed by MultiQC 1.2 aggregation to determine sequence quality, per-base sequence quality, per-read GC content (~50), and per base N content. Read pairs were aligned to the Bos taurus genome Ensembl build UMD 3.1 version 88 using STAR 2.5.2b employing a custom index, with read counting for an unstranded library preparation.
Data analysis	All algorithms and software used for RNAseq and ATACseq data analysis are published as indicated in the methods section. For RNAseq: Counts were normalized using Trimmed Means of M-values (TMM) as part of the edgeR package and modelled for biological and gene-wise variation. Differential expression between sample types was determined through the Exact Test in edgeR, with Benjamini-Hochberg multiple testing correction (FDR) set at < 0.05. For ATACseq: Raw ATAC-Seq reads were aligned to the bovine genome (Bostau 6) using Bowtie2. Aligned signals in raw (bam) files were filtered to remove PCR duplicates and reads that aligned to multiple locations. Peaks were identified using MACS v1.4.

For manuscripts utilizing custom algorithms or software that are central to the research but not yet described in published literature, software must be made available to editors/reviewers. We strongly encourage code deposition in a community repository (e.g. GitHub). See the Nature Research guidelines for submitting code & software for further information.

Data

Policy information about availability of data

All manuscripts must include a data availability statement. This statement should provide the following information, where applicable:

- Accession codes, unique identifiers, or web links for publicly available datasets
- A list of figures that have associated raw data
- A description of any restrictions on data availability

All primary RNA-Seq and ATAC-Seq data sets have been deposited with GEO.
To review GEO accession GSE132379:

Go to https://www.ncbi.nlm.nih.gov/geo/query/acc.cgi?acc=GSE132379
Enter token sfgtgoakdvidpaf into the box

Field-specific reporting

Please select the one below that is the best fit for your research. If you are not sure, read the appropriate sections before making your selection.

☒ Life sciences ☐ Behavioural & social sciences ☐ Ecological, evolutionary & environmental sciences

For a reference copy of the document with all sections, see nature.com/documents/nr-reporting-summary-flat.pdf

Life sciences study design

All studies must disclose on these points even when the disclosure is negative.

Sample size	Both RNA-Seq and ATAC-Seq data sets were acquired with two biological repeats to ensure the robustness of the data sets.
Data exclusions	No data was excluded
Replication	Both RNA-Seq and ATAC-Seq data sets were acquired with two biological repeats to ensure the robustness of the data sets. Each RT-qPCR or qPCR experiment was performed with 3-4 biological repeats and each RT-qPCR assay was performed with at least 2 technical repeats. Prism statistical software (GraphPad) was employed to analyze the data. In RT-qPCR experiments, gene expression was normalized to that of either Gapdh or Creb5, as indicated.
Randomization	Randomization was not relevant to this study.
Blinding	Blinding was not relevant as the data acquired is quantitative, and thus not subject to subjective interpretation

Reporting for specific materials, systems and methods

We require information from authors about some types of materials, experimental systems and methods used in many studies. Here, indicate whether each material, system or method listed is relevant to your study. If you are not sure if a list item applies to your research, read the appropriate section before selecting a response.

Materials & experimental systems

n/a	Involved in the study	
☐	☒	Antibodies
☐	☒	Eukaryotic cell lines
☒	☐	Palaeontology
☐	☒	Animals and other organisms
☒	☐	Human research participants
☒	☐	Clinical data

Methods

n/a	Involved in the study	
☒	☐	ChIP-seq
☒	☐	Flow cytometry
☒	☐	MRI-based neuroimaging

Antibodies

Antibodies used	Antibodies employed for Western Blots, ChIP, or immunocytochemistry are all listed in Supplemental Tables 6, 7, and 8.
Validation	Both catalogue number and vendors of Antibodies employed for Western Blots, ChIP, or immunocytochemistry are all listed in Supplemental Tables 6, 7, and 8.

Eukaryotic cell lines

Policy information about cell lines

Cell line source(s)	HEK 293T cells were used to package lentivirus. 293T/17 [HEK 293T/17] (ATCC® CRL-11268™) were obtained from ATCC. SW1353 cells were obtained from ATCC (HTB 94). The immortalized human costal chondrocyte cell line (C-28/I2)was obtained from Dr. Mary Goldring (Hospital for Special Surgery, Weill Cornell Medical College & Weill Cornell Graduate School of Medical Sciences).
Authentication	none
Mycoplasma contamination	cell lines were not tested for mycoplasma contamination.

Commonly misidentified lines (See ICLAC register)	not relevant

Animals and other organisms

Policy information about studies involving animals; ARRIVE guidelines recommended for reporting animal research

Laboratory animals	C57BL/6J mice were sacrificed at PO to obtain tissues for either Fluorescent In Situ Hybridization or immunocytochemistry Analysis.
Wild animals	N/A
Field-collected samples	The knee joints from 1-2 week old bovine calves were obtained from Research 87 (a local abattoir in Boylston, MA) directly after slaughter.
Ethics oversight	All work with mice has been approved by the Harvard Medical School IACUC.

Note that full information on the approval of the study protocol must also be provided in the manuscript.
